# Metabolic models predict fotemustine and the combination of eflornithine/rifamycin and adapalene/cannabidiol for the treatment of gliomas

**DOI:** 10.1093/bib/bbae199

**Published:** 2024-05-02

**Authors:** Ali Kishk, Maria Pires Pacheco, Tony Heurtaux, Thomas Sauter

**Affiliations:** Department of Life Sciences and Medicine, University of Luxembourg, L-4367 Belvaux, Luxembourg; Department of Life Sciences and Medicine, University of Luxembourg, L-4367 Belvaux, Luxembourg; Department of Life Sciences and Medicine, University of Luxembourg, L-4367 Belvaux, Luxembourg; Luxembourg Centre of Neuropathology, L-3555 Dudelange, Luxembourg; Department of Life Sciences and Medicine, University of Luxembourg, L-4367 Belvaux, Luxembourg

**Keywords:** glioma, metabolic modeling, drug repurposing, cancer

## Abstract

Gliomas are the most common type of malignant brain tumors, with glioblastoma multiforme (GBM) having a median survival of 15 months due to drug resistance and relapse. The treatment of gliomas relies on surgery, radiotherapy and chemotherapy. Only 12 anti-brain tumor chemotherapies (AntiBCs), mostly alkylating agents, have been approved so far. Glioma subtype–specific metabolic models were reconstructed to simulate metabolite exchanges, *in silico* knockouts and the prediction of drug and drug combinations for all three subtypes. The simulations were confronted with literature, high-throughput screenings (HTSs), xenograft and clinical trial data to validate the workflow and further prioritize the drug candidates. The three subtype models accurately displayed different degrees of dependencies toward glutamine and glutamate. Furthermore, 33 single drugs, mainly antimetabolites and TXNRD1-inhibitors, as well as 17 drug combinations were predicted as potential candidates for gliomas. Half of these drug candidates have been previously tested in HTSs. Half of the tested drug candidates reduce proliferation in cell lines and two-thirds in xenografts. Most combinations were predicted to be efficient for all three glioma types. However, eflornithine/rifamycin and cannabidiol/adapalene were predicted specifically for GBM and low-grade glioma, respectively. Most drug candidates had comparable efficiency in preclinical tests, cerebrospinal fluid bioavailability and mode-of-action to AntiBCs. However, fotemustine and valganciclovir alone and eflornithine and celecoxib in combination with AntiBCs improved the survival compared to AntiBCs in two-arms, phase I/II and higher glioma clinical trials. Our work highlights the potential of metabolic modeling in advancing glioma drug discovery, which accurately predicted metabolic vulnerabilities, repurposable drugs and combinations for the glioma subtypes.

## Graphical Abstract

**Graphical Abstract:**
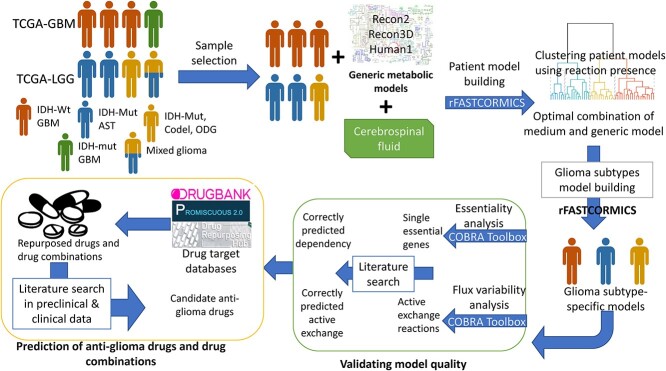

## INTRODUCTION

Gliomas account for 50% of the deaths in cases with primary malignant brain and central nervous system (CNS) tumors in the United States [1]. The 2021 World Health Organization (WHO) CNS classification [2] stratifies adult gliomas into three subtypes based on the mutation status of the isocitrate dehydrogenase 1/2 (IDH1/2) and the co-deletion of the short arm of chromosome 1 (1p) and the long arm of chromosome 19 (19q) (1p/19q co-deletion) into: astrocytoma (AST), IDH-mutant; oligodendroglioma (ODG), IDH-mutant and 1p/19q-codeletion ; and glioblastoma, IDH-wildtype (glioblastoma multiforme; GBM). GBM shows poor 7% 5-year survival that also limits the success rate of clinical trials, compared to 32%–53% in AST and 66%–84% in ODG [1].

The standard of care for GBM treatment is surgery, and radiotherapy, followed mainly by temozolomide (TMZ) chemotherapy [3]. Current approved anti-brain chemotherapies (AntiBCs) consist of eight monotherapies (cell cycle inhibitors and anti-hypoxic agents) and two combinations: procarbazine/lomustine/vincristine (PCV) and dabrafenib/trametinib [4]. The monotherapy AntiBCs targeting the cell cycle are mostly alkylating agents (TMZ, lomustine, carmustine and cyclophosphamide) and only doxorubicin targets topoisomerase; meanwhile, three anti-hypoxic agents reduce angiogenesis by targeting the mTOR/HIF-1α/VEGF pathway: everolimus (mTOR), belzutifan (HIF-1α) and bevacizumab (VEGF). The recently approved dabrafenib/trametinib combination for BRAF-mutant low-grade glioma (LGG) is the only targeted ABC inhibiting the RAF/MEK pathway. Whether targeting the RAF/MEK by dabrafenib/trametinib or the cell cycle by PCV combination, both combinations show redundancy of the target pathways, which is another potential cause for inefficacy of positive phase II drugs in phase III GBM trials [5]. Despite these treatments, glioma patient survival stays poor, with a high recurrence rate. The need for efficacious drugs and combinations targeting alternative pathways is therefore pivotal.

Drug repurposing, i.e. redirecting approved drugs to other diseases, has been proven as a critical element to shorten the lengthy toxicity trials in cancer drug discovery. However, current preclinical drug repurposing approaches in glioma have been mainly limited to GBM [6, 7], with a high failure rate in clinical trials due to non-efficacy, poor cerebrospinal fluid (CSF) bioavailability, drug resistance and toxicity [3]. While the exact biological role of 1p/19 co-deletion is still unclear, IDH mutation in most LGG dysregulates the nicotinamide adenine dinucleotide phosphate (NADPH) balance and glutamate biosynthesis, depleting the glutathione, activating oxidative metabolism and increasing the reactive oxygen species (ROS) sensitivity [6].

The role of metabolic rewiring in IDH-mutant glioma encouraged the use of metabolic modeling for the study of gliomas, notably AST and ODG. Metabolic modeling is commonly applied to model the metabolism of cancer cells and to select among all FDA-approved drugs, the ones that target specifically cancer vulnerabilities appearing from metabolic rewiring [8]. Whole-brain and brain cell models were reconstructed to study alterations in the metabolism in neurodegenerative disease and GBM. These published genome-scale brain metabolic models were extensively covered in our previous review [9]. In the present study, we reconstructed three glioma subtype models using patient data from the TCGA, predicted drug and drug combinations, as well as the predicted essential genes in the different subtypes. Extensive literature review and comparison against high-throughput screenings (HTSs), especially against AntiBCs, allowed confirming the model’s prediction and further prioritizing the drug candidates.

### Importance of the study

Due to high relapse and drug resistance rates, the three glioma subtypes suffer from poor survival rate, calling for new therapies. We present genome-scale metabolic models (GEMs) of the three well-defined glioma subtypes that predicted repurposable FDA-approved single drugs and combinations for gliomas. We confirmed our predicted drugs using published *in vitro* and xenograft drug screenings and found that antimetabolites and TXNRD1-inhibitors induced a growth reduction comparable to AntiBCs *in vitro* and in xenografts. Furthermore, fotemustine showed a higher effectiveness in GBM clinical trials than AntiBCs. Additionally, we predicted 17 drug combinations mostly to be efficient on all three subtypes, with eflornithine/rifamycin and cannabidiol/adapalene being GBM- and LGG-specific, which is coherent with the known LGG-specific glutamate and glutathione depletion. This work presents the first GEMs that go beyond glioblastoma into the glioma subtypes, accurately capture the intra-heterogeneity and further predict repurposable combinations.

## MATERIALS AND METHODS

### Model building

Two types of models were built using rFASTCORMICS [10]: sample models to assess the subtype intra- and inter-heterogeneity and consensus models for the three glioma subtypes used for essential gene and drug prediction (see Supplementary Methods for more details). rFASTCORMICS was thereby fed with RNA-Seq data (116 GBM samples and 257 LGG samples) from The Cancer Genome Atlas Program (TCGA) data [11], stratified based on the 2021 WHO CNS classification [2], with the generic model Recon3D [12] as input reconstruction and the composition of CSF [13] as medium constraint. Other models and data formats were tested, but this setting allowed better separation between the sample models of the three glioma subtypes (Supplementary Methods, Figures S1–S4 and Table S1), matching to literature-retrieved metabolic exchanges (Figure S5) and balanced capturing of common essential genes (Figure S6). To confirm the models’ predictions in terms of metabolite exchanges, essential gene and drug predictions were compared with literature and databases, notably DepMap [14].

### Prediction of metabolite exchanges

Different uptake and release reactions in the glioma subtype models allowed for assessing the model quality by comparing the exchange reactions with literature evidence. The minimum and maximum fluxes for the input and output reactions of the three consensus subtype models were computed using the *fluxVariability* function of the COBRA Toolbox v.3.0 [15] while maximizing for biomass production (biomass reaction). Narrow-bounded exchange reactions were selected as any perturbation is predicted to alter the cell growth of the models.

### Prediction of essential genes

Single gene deletion from the COBRA Toolbox v.3.0 [15] was used with biomass optimization to predict essential genes. Only genes whose knockout (KO) is predicted to reduce the growth by at least 50% were selected and compared to a list of common essential genes (defined by the Cancer Dependency Map, DepMap, as genes found to be essential in >90% of cell lines in pan-cancer CRISPR-Cas9 screens), retrieved from DepMap 22Q1 [14].

### Prediction of anti-glioma drugs and drug combinations

To predict potential single drugs and drug combinations, the drug deletion pipeline [16] was run and hence every target of the FDA-approved drugs and drug combinations was knocked out to assess the predicted effect on cell growth. Single drugs were restricted to FDA-approved drugs (2387 drugs defined by Drug Repurposing Hub [17]) due to the high failure rate in glioma clinical trials of preclinical compounds. For the drug combinations, AntiBCs and investigational anti-glioma drugs (IAGs) were tested in concert with FDA-approved drugs. Only single drugs and combinations that reduced the growth by at least 50% were considered for further analysis. Single drugs predicted to shut down the biomass production completely were not further tested in combination with other drugs. The drug targets were retrieved from DrugBank [18], PROMISCUOUS2 [19] and Drug Repurposing Hub [17]. Information on the IAGs (41 drugs) was gathered from the orpha.net database (ORPHA:182067), and AntiBCs (12 drugs) were retrieved from a review [4] and the NIH website (https://www.cancer.gov/about-cancer/treatment/drugs/brain).

### Drug prioritization and benchmarking

Different clinical, xenograft, *in vitro* and pharmacokinetics (PK) data in brain cancer were collected to rank the predicted drugs based on their efficacy (see Supplementary Methods for details) and classify them into effective, ineffective and untested. Most compiled clinical trial data were on phase I/II or higher clinical trials in brain cancer (*n* = 50) and two-arm trials in glioma (*n* = 8). Two metrics were considered: overall survival (OS) and progression-free survival (PFS, duration between treatment and symptom worsening). The xenograft data included *in/ex vivo* drug HTSs in GBM patient-derived xenografts (PDXs) and *in vivo* drug screening from literature. *In vitro* data combined two cellular metrics: IC_50_ and viability reduction, and hence, a median IC_50_ across brain cancer cell lines was calculated for each drug as a potency measure. If available, PK data corresponding to CSF bioavailability was prioritized over blood–brain barrier (BBB) permeability as the latter cannot capture the efflux rates of the brain. Single drugs that induced proliferation *in vitro* or cofactors to the target genes were excluded from further ranking. CSF bioavailability data were collected as *LogBB* (logarithm of the drug’s CSF-to-plasma concentration ratio).

## RESULTS

Glioma sample models were reconstructed from TCGA-GBM and TCGA-LGG to assess if the metabolism of the 2021 WHO classification glioma subtypes was sufficiently different to be captured by qualitative metabolic models. The subtype models (iGBM, iAST, iODG) include between 32% and 35% of the reactions of the generic metabolic reconstruction Recon3D (Table 1), accurately detected metabolic variations between the IDH-mutant and -wildtype samples and allow for a clear separation between both types (Figure S3).

**Table 1:** Summary of selected samples and model statistics for the consensus glioma subtype and control models

Model	Selected samples	Reactions	Metabolites	ENTREZ Genes
iAST	116	3269	2460	1273
iGBM	140	3526	2621	1302
iODG	117	3331	2504	1305
iCTRL	4	3407	2585	1603

### LGG and GBM models correctly predict high glutamate and thymidine uptake rates

**Figure 1:**
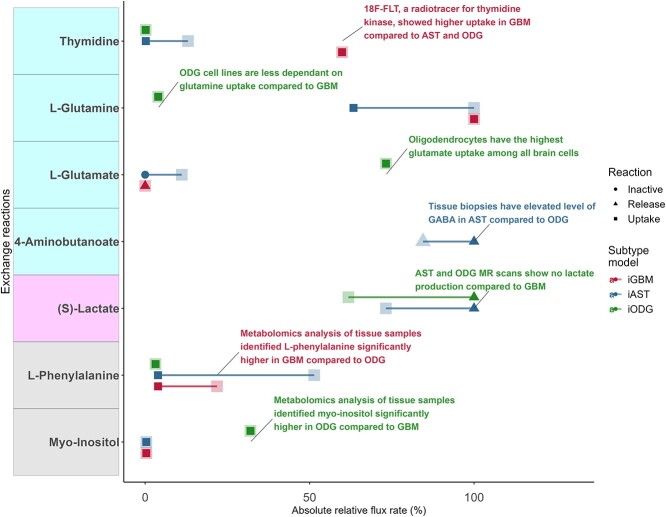
The predicted input and release rates of metabolites match the literature and experimental observations. Flux Variability Analysis allowed calculating flux ranges that guarantee an optimal growth and hence allowed finding critical metabolite exchanges (narrow-bounded fluxes). Forty out of 101 predicted exchanges were narrow-bounded, and four of the five exchange reactions matched literature and data from radiotracers for three subtypes (see Figure S7 for all reactions). Two exchange reactions matching metabolomics data were considered minor validation due to the unclear metabolic flux. Metabolite names on the *y*-axis with cyan, magenta and gray correspond to literature matching, contrary to literature and minor validation (Table S2), respectively.

Input and release rates of metabolites were predicted for the three consensus subtype models and compared to the literature (Table S2). Forty metabolites of the predicted 101 metabolite exchanges had narrow-bounded fluxes (with a maximum of 10% of the maximal range) and are predicted to affect cell growth directly. Four of them (Figure S7) matched known differences between the subtypes in cell line uptake, patient biomarkers and MR radiotracers (Figure 1, in cyan). High glutamate uptake was predicted in the LGG models (iAST and iODG), concordant with the known glutamate depletion due to IDH-mutant-induced rewiring [20]. iGBM predicted the highest thymidine uptake, in agreement with an elevated [18]F-FLT radiotracer uptake in GBM patients’ scans compared to AST [21] and ODG [22]. Finally, the reduced glutamine uptake in iODG conforms to the low ODG-specific glutamine dependency [23]. Together, predicted glutamate and thymidine uptake variations followed known IDH-based differences in the three subtypes. In one instance, the predicted lactate exchange is inconsistent with the literature [24] (Figure 1, in magenta). The lactate exchange inconsistency could be attributed to the inactivity of lactate exchange reactions during iGBM model building and their exclusion from the consistent subnetwork. Meanwhile, variations within the subtypes matching metabolomics data in L-phenylalanine and myo-inositol were considered minor validations (Figure 1, in gray). For the remaining exchanges, no data could be found in the literature. Notably, octadecenoate and pyruvate could serve as potential biomarkers between the glioma subtypes, but they still require validation (Figure S7). These experiments were repeated with 90% and 95% of maximation. However, the lowering of the threshold turned most exchanges to become unbounded, due to the high degree of freedom and hence could no longer capture the observations gathered from the literature. For example, glutamine exchange in iGBM was irreversible with 90% maximization, wide uptake range with 95% and high narrow-bounded with 100% maximization. 100% maximization of glutamine exchange in iGBM was the only setting matching high glutamine uptake in GBM (Figure S8).

Taking together, the metabolic models mostly captured not only metabolic variations between the subtypes but also recapitulated experimental observations.

### Thioredoxin detoxification and nucleotide interconversion are potential targets for all three subtypes and arginine uptake for GBM

**Figure 2:**
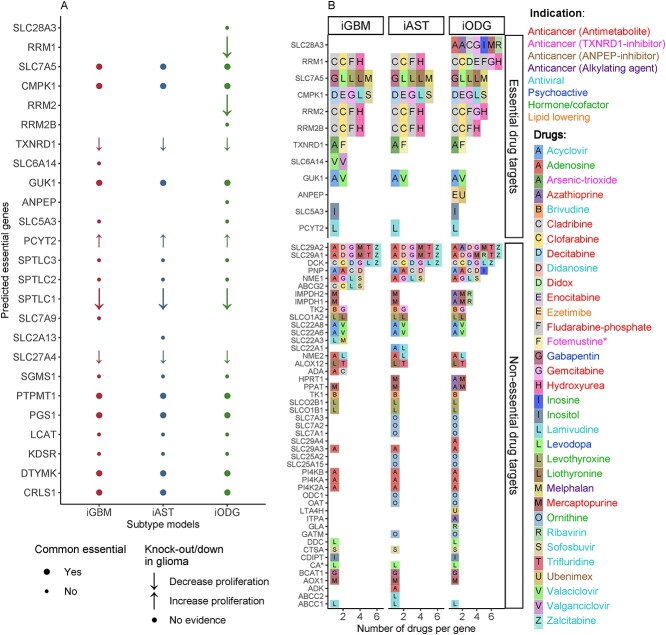
Thioredoxin detoxification and nucleotide interconversion are predicted as potential drug targets for glioma. (**A**) Twenty-five genes were predicted to reduce tumor growth *in silico* KO. Six of the 25 essential genes have been previously tested in *in vitro* KO or KD studies glioma (literature, Table S3), where five genes lowered proliferation (down arrows), and only one (PCYT2) increased proliferation (up arrows). Genes with no support in the literature are marked as dots. Moreover, 10 of the 25 predicted essential genes are common essentials (found essential genes in most cancer cell lines from CRISPR screens) and are marked as a big dot or arrow in (**A**). (**B**) Thirty-three FDA-approved drugs were predicted to reduce cell growth and could hence be repurposable for glioma. The 33 single drugs have 60 targets, of which 12 are essential genes (‘Essential drug targets’). Classifying the drugs based on approved indication and mode-of-action (MOA) (see color code of the font) showed that nearly a third are antimetabolites and fotemustine (with *) has both TXNRD1-inhibitor and alkylating MOA. For example, in (**B**), cladribine is marked as ‘C’ letter in gray box, while its approved indication and MOA is represented in red in the drug name color.

We further predicted vulnerabilities (essential genes) that could be exploited to reduce tumor progression. Twenty-five genes were predicted to be essential (Figure 2A) with 100% growth reduction (Figure S9). iODG yielded the highest number (*n* = 22), matching its known high survival rate and vulnerability [25]. Ten of these genes were identified as common essential genes by DepMap [14], suggesting pan-cancer vulnerabilities. A literature search further found five genes (TXNRD1, RRM1–2, SPLTC1, SLC27A4) to reduce viability with *in vitro* knockdown (KD) or KO. The KO of thioredoxin reductase (TXNRD1) reduced proliferation and migration in drug-resistant GBM [26]. Moreover, TXNRD1 expression significantly correlated with poorer diagnosis in AST [27] and ODG [28] patients. Due to their radical-scavenging activities, thioredoxin and glutathione (GSH) control oxidative homeostasis and counter mitochondrial oxidative stress. Similarly, KD of SPLTC genes involved in sphingolipid synthesis reduced the viability of GBM cell lines [29]. Likewise, the KD of RRM1 and RRM2, involved in nucleotide interconversion, caused cell death and sensitized GBM to TMZ, respectively (Table S3). However, one predicted essential gene: PYCT2, was described to increase GBM proliferation in a KD experiment [30].

Besides the potential vulnerabilities, 33 FDA-approved drugs could, according to our models, be considered repurposable for glioma (Table S8). The 33 predicted drugs include 14 non-brain anti-cancer drugs (anticancers), 10 antivirals, 6 hormones/cofactors, 2 psychoactives and 1 lipid-lowering agent. The 14 anticancers are 10 antimetabolites, an ANPEP-inhibitor, 2 TXNRD1-inhibitors (such as fotemustine, also being an alkylating agent) and an alkylating agent. These drugs target 12 essential genes and 48 non-essential genes (Figure 2). Among them, TXNRD1 is targeted by arsenic trioxide and fotemustine (approved in some countries against melanoma brain metastasis [31]), and RRM1-2 by seven antimetabolites. RRM1-2 showed higher dependency probability (the likelihood that the KO of a gene reduces cell growth or induces cell death) than AntiBCs’ targets in the glioma cell lines (Figure S10). Furthermore, we predicted valganciclovir that affects arginine transporter SLC6A14 as a GBM-specific single drug.

The drug target genes differ among the three subtypes for the same drugs due to differences between the subtype models in gene and reaction compositions during model building. Some of the various targets of several drugs were included in some models and excluded in other based on the expression data*.* The glioma subtype model genes were compared to other brain metabolic models discussed in our previous review [9] using the Human Protein Atlas [32] brain-specific gene categories. The glioma subtype models showed comparable completeness and specificity to curated and semi-curated brain metabolic models (Figure S11).

Taken together, metabolic modelling accurately captured pan-glioma single vulnerabilities, such as thioredoxin detoxification and nucleotide interconversion. Additionally, metabolic modelling proposes arginine uptake as a druggable vulnerability for GBM.

### Glutamate and polyamine biosynthesis are predicted suitable target pathways for drug combinations in LGG and GBM, respectively

**Figure 3:**
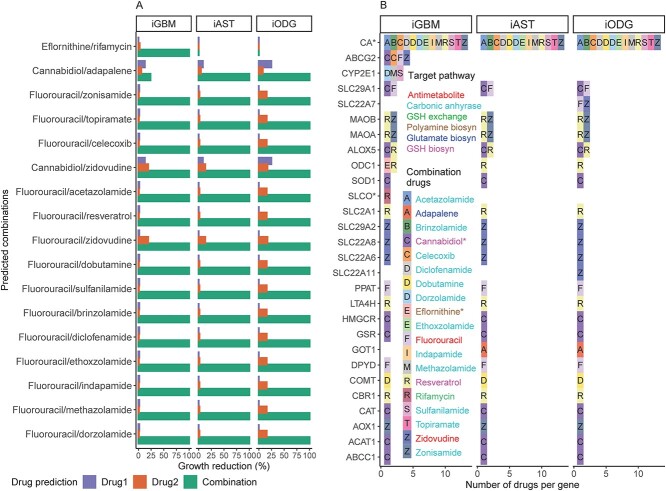
Eflornithine/rifamycin and cannabidiol/adapalene are predicted safe synergistic combinations for GBM and LGG, respectively. (**A**) Drug combination predictions were performed between two drug sets: (**a**) FDA-approved drugs after excluding predicted single drugs with a predicted lethal on the models and (**b**) approved AntiBCs and IAGs (marked with *). Combinations with growth reduction above 50% are depicted. All combinations, but two, reduce tumor growth across glioma subtypes (top two). (**B**) Analysis of the targets from the 17 synergistic combinations showed that dual KO of ODC1-SLCO (SLCO1A2, SLCO1B1, SLCO2A1 and SLCO2B1) genes are GBM-specific, while GOT1 and cannabidiol targets are LGG-specific. The drug names are colored based on the targeted pathway. Abbreviations: GSH, glutathione; biosyn, biosynthesis.

FDA-approved drugs were tested *in silico* in combination with a set of 53 AntiBCs and IAGs to find meaningful synergistic drug combinations. Seventeen combinations (Figure 3A) composed of 19 drugs (hereafter will be referred to as combination drugs), including one anticancer antimetabolite (fluorouracil), antiviral antimetabolite (zidovudine), 13 carbonic anhydrase inhibitors (CAi) and two herbal antioxidants (cannabidiol and resveratrol) with multi-target actions (Table S4 and Tables S9–10). As every two drugs in the combinations have independent targets, Bliss combination index was selected to find synergistic, antagonistic and additive combinations [33] using growth reduction (1-grRatio, see Materials and Methods for details) as drug effect. Fifteen combinations with CAi were predicted to be synergistic in the three subtypes. CA converts CO_2_ to bicarbonate and is matching the known anti-glioma action of CAi by decreasing extracellular acidosis responsible for drug resistance. Besides CA, zonisamide and resveratrol target MAOA and MOAB genes, which convert oxygen and water into hydrogen peroxide, thereby increasing intracellular hypoxia, with MAOA inhibition found to decrease glioma proliferation and angiogenesis [34]. Two combinations displayed subtype-specific synergism (eflornithine/rifamycin for GBM and cannabidiol/adapalene for LGG), achieved 100% growth reduction their corresponding subtypes and did not affect ATP production and biomass maintenance in the healthy model iCTRL (Figure S12). Eflornithine (also known as α-difluoromethylornithine or DFMO) inhibits ornithine decarboxylase (ODC1), coding for an enzyme of the polyamine biosynthesis pathway, while rifamycin targets SLCO genes associated with GSH exchange reduction (Figure 3B). Meanwhile, adapalene inhibits glutamic-oxaloacetic transaminase 1 (GOT1) that governs glutamate biosynthesis from alpha-ketoglutarate, while cannabidiol increases ROS by depleting glutathione production. Both glutamate and GSH biosynthesis depletion align with the known LGG-specific vulnerabilities [20]. Among the combinations drug targets, ABCC1 and ABCG2 of the ATP-binding cassette (ABC) transporters predicted to remove the toxic byproducts of lipid peroxidation (4-hydroxy-2-nonenal) and heme biosynthesis (protoporphyrin), respectively. The predicted GBM-specific protoporphyrin is consistent with impaired heme biosynthesis in LGG cell line [35]. Similarly, 4-hydroxy-2-nonenal was detected in GBM and AST samples affirming the predicted pan-glioma profile of the lipid peroxidation [36]. Altogether, metabolic modeling predicted combinations targeting alternative reactions for potential synergism, many of these reactions match known subtype-specific biosynthesis vulnerabilities. Of these combinations, the combined targeting of glutamate and GSH biosynthesis is a potentially druggable combination in LGG; meanwhile, targeting polyamine synthesis combined with GSH exchange is a potentially druggable combination in GBM.

### Gemcitabine, cladribine and decitabine have better CSF bioavailability and *in vitro* potency than AntiBCs

**Figure 4:**
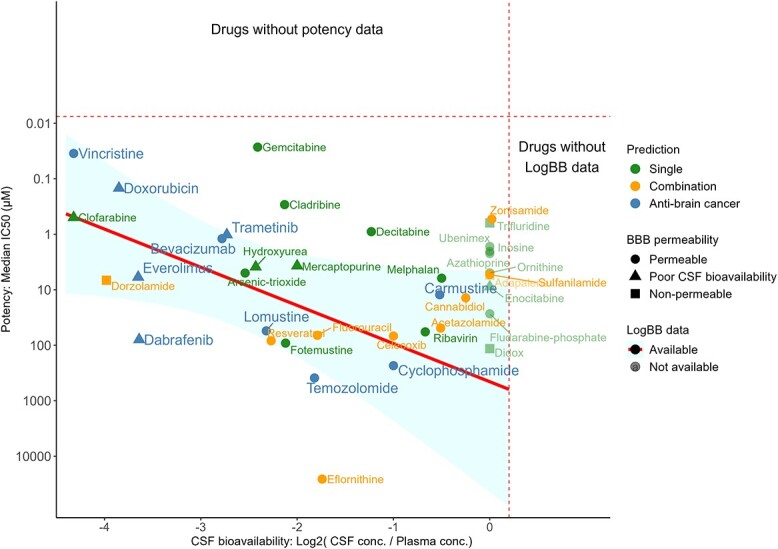
Gemcitabine, cladribine and decitabine showed stronger *in vitro* potency and CSF bioavailability than AntiBCs. Potency (*y*-axis) and CSF bioavailability (*x*-axis) data were collected from the literature and screening databases (see Figure S13 for detailed potency per database). Potency (median IC_50_ across the brain cancer cell lines) was calculated for single and combination drugs and the AntiBCs. Similarly, CSF bioavailability was collected in *LogBB*, which is the logarithm of the CSF-to-plasma concentration ratio. Rightward on the *x*-axis and upward on the *y*-axis represent increasing potency and CSF bioavailability, respectively. Drugs without potency or *LogBB* data are on the top and right sides separated by the dashed lines, respectively.

To select the most promising drugs and drug combinations, we ranked the drugs from HTS and literature data using IC_50_, viability reduction, BBB permeability, CSF bioavailability, ABC transporter affinity, *in/ex vivo* xenograft testing, main MOA, phase I/II clinical trials or higher and possible drug–drug interactions, aggregated from HTS and the literature (Supplementary File 2, Tables S8–S16, Supplementary Methods on data gathering and drug ranking and Table S5 for the screening databases). Ten single drugs were excluded for being cofactors to the target genes or induced proliferation *in vitro* (Table S8). As expected, the CSF bioavailability of AntiBCs is inversely correlated with potency (Figure 4 and Figure S13). Many single drugs, such as fotemustine, arsenic trioxide and hydroxyurea, showed comparable balanced outcomes to AntiBCs. In contrast, three antimetabolites (decitabine, gemcitabine and cladribine) achieved good potency and bioavailability. These three antimetabolites, especially gemcitabine, notably reduced cell viability compared to most AntiBCs (Figure S14). Some drugs showing high potency, such as clofarabine (predicted) and doxorubicin (AntiBC), however, had poor bioavailability. Taken together, most drug candidates achieved comparable results (TXNRD1-inhibitors) or outperformed (antimetabolites) AntiBCs drugs in terms of potency and bioavailability.

### Cladribine and clofarabine reduced growth in GBM PDXs, while fotemustine reduced xenograft growth (data from literature)

**Figure 5:**
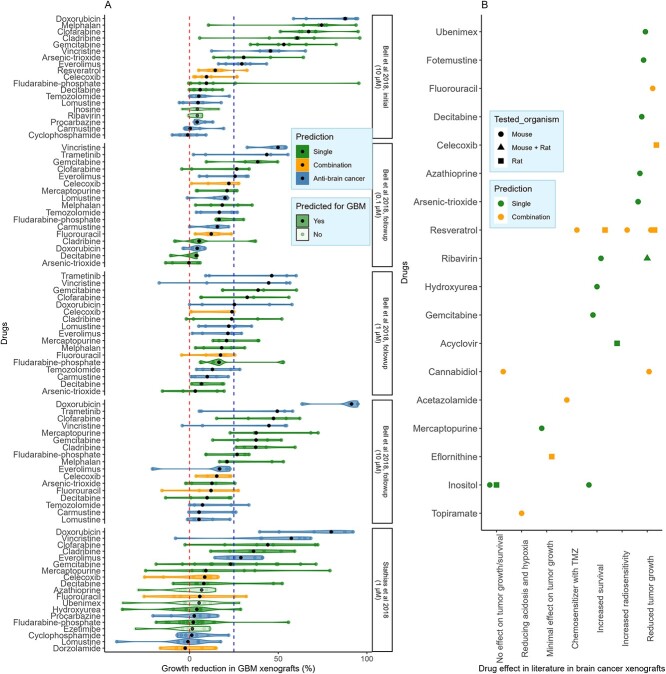
Clofarabine has a more robust growth reduction than two-thirds of the AntiBCs in GBM PDXs. Xenograft data were collected from HTS tested in GBM PDXs (**A**) and literature tested in brain cancer generally (**B**). Clofarabine, gemcitabine and cladribine attained a more robust or comparable growth reduction than half of the AntiBCs in the HTS. Additionally, some drugs not predicted by iGBM (in light green) showed moderate growth reduction in the GBM PDXs.

Three drug HTS in GBM PDXs [37, 38] (Figure 5A) and PDXs data from the literature (Figure 5B and Table S12) were used to test the drugs in biological contexts closer to *in viv*o. Four non-alkylating AntiBCs (vincristine, trametinib, everolimus and doxorubicin) (Figure 4) surpassed 25% PDXs growth reduction. Correspondingly, three antimetabolites (gemcitabine, cladribine and clofarabine) induced a growth reduction comparable to or higher than these four AntiBCs. However, decitabine showed non-conclusive results between the drug screenings and the literature (Table S12). Fotemustine, which was not tested in HTS PDXs experiments, reduced growth *in vivo* in literature [39]. However, several drugs that showed low growth reductions in the GBM PDXs were predicted only by the LGG subtypes consensus models. Four combination drugs (fluorouracil, celecoxib, resveratrol and acetazolamide) (Tables S12 and S13) sensitized glioma to TMZ *in vitro* and *in vivo*. Of these, celecoxib caused a moderate *in vivo* growth reduction (with a median of 9%–22%). In summary, the three antimetabolites presented steady *in vitro* potency and *in vivo* growth reduction, and clofarabine outperformed two-thirds of the AntiBCs in PDXs.

### Fotemustine alone and eflornithine, celecoxib and valganciclovir in combination improved median OS compared to AntiBCs in phase II glioma trials, while antimetabolites showed no improvement

**Figure 6:**
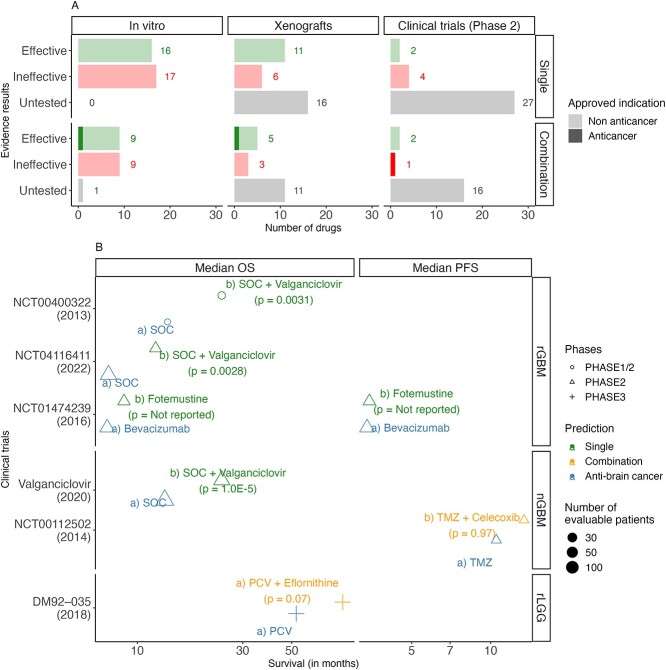
Among the tested predicted drugs, half and two-thirds were effective *in vitro* and xenografts, respectively, with four drugs showing comparable survival to AntiBCs in two-arm, phase I\II clinical trials. (**A**) Predicted single and combination drugs were classified into effective, ineffective and untested against AntiBCs using the criteria in Table S5. (**B**) Two-arm, phase II clinical trials in gliomas were selected to compare the predicted drugs to the ABC arm as monotherapy or in combination. Among the single drugs, fotemustine monotherapy and valganciclovir in combination improved median OS. Likewise, eflornithine and celecoxib improved mOS and median PFS, respectively. Only valganciclovir achieved statistical significance versus the ABC arm. On the other hand, two antimetabolites (gemcitabine and cladribine) and melphalan failed in single-arm trials with no reported survival, while mercaptopurine and fluorouracil reduced and kept mOS when combined with carmustine, respectively. Statistical significance of the predicted drug arm versus the AntiBCs arm was reported independently for each trial. Abbreviations: rGBM: recurrent GBM, nGBM: newly diagnosed GBM, rLGG: recurrent LGG, TMZ: temozolomide, SOC: standard-of-care, PCV: Procarbazine/lomustine/vincristine combination.

Drugs were classified into effective, ineffective and untested for *in vitro* and xenografts using the criteria in Table S6. All single and combination drugs except rifamycin were tested *in vitro*, while only half were tested in xenografts. Of the tested drugs, half and two-thirds were found effective in *in vitro* and xenografts, respectively (Figure 6A). Among the two-arm, phase I/II trials (Table S11), two single drugs (fotemustine and valganciclovir) and two combination drugs (eflornithine and celecoxib) improved the primary survival outcome compared to the ABC arm (Figure 6B). On the other hand, three predicted single drugs show no or minimal activity as monotherapy in single-arm, phase II trials: gemcitabine [40] (AST and GBM), cladribine [41] (AST and ODG) and melphalan [42] (AST and GBM). Additionally, when combined with carmustine, mercaptopurine and fluorouracil showed an antagonistic [43] and a non-additional [44] effect, respectively. The five drugs which failed in phase II trials are either substrate or inducer to the ABC transporters (Table S15), which might cause drug resistance, and all are cell cycle inhibitors.

Beyond cell cycle inhibitors, of the four clinically effective drugs (fotemustine, valganciclovir, eflornithine and celecoxib), only valganciclovir reached statistical significance in the primary outcome in newly diagnosed GBM (nGBM) [45] and recurrent GBM (rGBM) [46, 47]. After the first-line treatment of TMZ and radiotherapy, fotemustine monotherapy slightly exceeded bevacizumab by 1.3 months achieving 8.7 months median OS (mOS) in a phase II trial in rGBM [48]. Eflornithine added to the PCV combination improved the mOS in recurrent LGG with about two and a half years against the PCV combination alone [49]. To a lesser extent, celecoxib combined with TMZ increased median PFS by 3 months in nGBM versus TMZ alone [50] and is the only known ABC transporter inhibitor of the four clinically effective drugs. While none of the 17 predicted combinations had been tested in brain cancer, three combinations (fluorouracil/zidovudine, fluorouracil/celecoxib and fluorouracil/resveratrol) were found synergistic in non-brain cancer *in vitro*; however, the last two failed as a combination in various clinical trials (Table S7). Eflornithine/rifamycin and cannabidiol/adapalene were ranked first and second for GBM- and LGG-specific subtypes, respectively. Despite the clinical activity of celecoxib in GBM, fluorouracil/celecoxib was ranked fourth due to predicted major drug–drug interaction (DDI) by DrugBank (Table S16) and the absence of an additional effect of the combination in phase III colon cancer trial [51]. Meanwhile, zonisamide showed the strongest *in vitro* potency among the combination drugs, and the fluorouracil/zonisamide was predicted with minor DDI of increased arrhythmia; hence, this combination was ranked third. All in all, metabolic modeling predicted drug candidates with a steady effective-to-ineffective ratio in *in vitro* (49%), in xenografts (64%) and in clinical trials (44%). Unlike the redundancy of the AntiBCs combinations’ target pathways, predicted combinations covered multiple alternative pathways, increasing potential synergism, of which three combinations were tested in non-brain cancer.

## DISCUSSION

We have built GEMs (iGBM, iAST and iODG) for the three glioma subtypes based on the 2021 WHO classification and have predicted new repurposable, single drugs and combinations. While the sensitivity of essential gene predictions of the metabolic models remains low, the specificity is high [52, 53]. The low sensitivity is often attributed to the fact that *in silico* single gene KOs only capture essentiality related to metabolism and most specifically to the optimization function and fail capturing essentiality to other processes such as regulatory processes. The large efforts invested in the curation and standardization of genome-scale metabolic reconstruction, notably MEMOTE [54] and MetaNetX [55] as well as the benchmarking of context-specific model algorithms [56–58], and the curation of GPR rules [59] and improvement of biomass formulation [53] are likely to further improve the accuracy of the predictions [52, 60]. Furthermore, unlike some other computational drug repurposing approaches, metabolic modeling allowed understanding the effect of a KD or KO on a system level and confronting it to the earlier knowledge. Various sanity checks allowed for biologically relevant predictions that included: model selection based on subtype separation, matching metabolic exchanges and gene KD/KO with literature and evaluation against AntiBCs *in vitro*, in xenografts and in clinical trials. While model-building used the rFASTCORMICS algorithm [10], a member of the FASTCORE family that were benchmarked by us and others in various studies [57, 58], data collected of gene KD/KO and metabolic exchanges were kept only for validations as recommended in this review [61]. The models recapitulated metabolite exchanges and subtype-specific uptake for radiotracers in patients and medium metabolites measured *in vitro*. Similarly, IDH-mutant models (iAST and iODG) accurately predicted vulnerabilities consistent with the known 2HG-induced NADPH depletion, such as targeting glutamate and GSH biosynthesis [20] with the cannabidiol/adapalene combination, which would aggravate the depletion.

**Figure 7:**
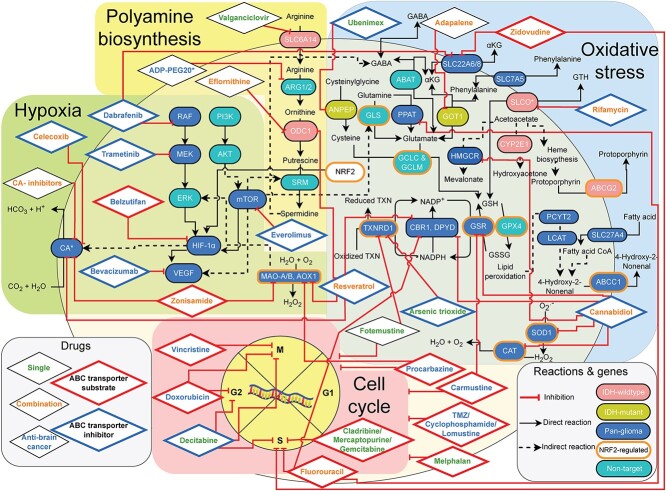
Predicted drugs target the same genes as the AntiBCs or downstream genes, mainly covering four biological pathways: hypoxia, oxidative stress, cell cycle and polyamine biosynthesis. AntiBCs mainly affect hypoxia and cell cycle via growth factors and unselective alkylation, respectively. Meanwhile, the predicted CAis and antimetabolites selectively target DNA biosynthesis and CA, respectively. The transcription factor NRF2, a target of AntiBCs, regulates a third of predicted targets in the oxidative stress pathway (in olive green) [64–66]. Furthermore, predicted drugs and AntiBCs share common targets such as glutathione reductase (GSR) between cannabidiol and carmustine, monoamine oxidase A/B (MAO-A/B) are common between procarbazine, zonisamide and resveratrol and SLC22A6/8 between dabrafenib and zidovudine. Most predicted drugs and cell cycle inhibitor AntiBCs that were shown to be clinically inefficient are ABC transporter substrates, while clinically effective predicted drugs are either non-substrate (eflornithine), inhibitor (celecoxib) or have an unknown effect (fotemustine and valganciclovir) on ABC transporter. Unlike clinically ineffective antimetabolites (cladribine, gemcitabine, mercaptopurine and fluorouracil), fotemustine targets both cell cycle and TXNRD1. Meanwhile, polyamine synthesis is targeted uniquely by the clinically effective drugs predicted for GBM (valganciclovir and eflornithine), the phase I/II ADPI-PEG20 IAG. Furthermore, cannabidiol, rifamycin, celecoxib and others inhibit the ABC transporters (ABCC1 and ABCG2) responsible for AntiBCs resistance as well as byproducts detoxification such as the efflux of lipid peroxidation (4-hydroxy-2-nonenal) and heme biosynthesis (protoporphyrin) byproducts. 4-hydroxy-2-nonenal and protoporphyrin predicted for pan-glioma and GBM, respectively, matching the literature in the predicted subtypes. Abbreviations: GSH: reduced glutathione, GSSG: oxidized glutathione, TXN: thioredoxin, αKG: alpha-ketoglutarate.

Cell cycle and hypoxia are the two common target pathways for glioma chemotherapy, with both AntiBCs combinations targeting redundant pathways diminishing a potential synergism. However, cell cycle inhibitors are ABC transporter substrates and hence are transporting the drugs out of the tumor causing drug resistance [62, 63] (Figure 7). Meanwhile all anti-hypoxic AntiBCs, except belzutifan, are ABC transporter inhibitors. Despite poor CSF bioavailability, only non-alkylating AntiBCs surpassed 25% growth reduction in xenograft experiments at 10 μM or lower, which is coherent with the mediocre performance in improving the OS in glioma patients. The subtype models allowed predicting single drugs and drug combinations that could enlarge the panel of therapeutic options in glioma. The single drug candidates predicted in this study principally target oxidative stress through the KD of TXNRD1, and arginine uptake and nucleotide interconversion (antimetabolites).

TXNRD1 is predicted to be a vulnerability present across all glioma subtypes, and its expression was shown to highly correlate to AST [27] and ODG [28] patients’ survival. Moreover, fotemustine (TXNRD1-inhibitor and alkylating agent) monotherapy displayed comparable survival to bevacizumab in a two-arm, phase II clinical trial in rGBM [48]. In a network meta-analysis of 11 AntiBCs and IAGs in rGBM, fotemustine ranked best in effectiveness as mOS [67]. Another TXNRD1-inhibitor, arsenic trioxide, was tested as local interstitial monotherapy in a single-arm, phase I/II GBM trial with the first promising results [68], outperformed alkylating AntiBCs *in vivo* and *in vitro* and an ABC transporter inhibitor.

The antiviral valganciclovir, one of our drug candidates, is predicted to target arginine uptake in GBM. *In vitro*, arginine deprivation reduced GBM invasiveness [69] and currently, the arginine-degrading agent, ADI-PEG20, has been tested in a phase I GBM trial (NCT04587830) [70]. While valganciclovir significantly enhanced mOS when combined with the AntiBCs in both nGBM and rGBM phase II trials, its hypothetical cytotoxic link to arginine uptake inhibition has yet to be proven.

While antimetabolites have been successful in cancer treatment and despite the high *in vitro* potency and viability reduction, four antimetabolites (cladribine, gemcitabine and mercaptopurine from the single drugs and fluorouracil from the combinations drugs) failed in phase II glioma trials. Another predicted antimetabolite, clofarabine, showed *in vivo* growth reduction, but has not been tested clinically. While BBB permeability and CSF bioavailability infer drug penetrance, ABC transporter affinity was found to be more crucial for drug diffusion into core tumor regions, even under leaky BBB [71]. Similarly, ABC transporter substrates are more likely to possess low serum concentration due to drug metabolism. Both clinically ineffective predicted drugs and cell cycle inhibitors AntiBCs are ABC transporter substrates, which might explain their inefficacy to due to limited drug distribution to the core tumor [71]. Finally, despite having the highest potency, CSF bioavailability and ABC transporter inhibitor affinity, decitabine presented moderate *in vitro* viability and weak xenograft growth reduction. Nevertheless, decitabine prodrugs are currently in two phase II trials for IDH-mutant glioma (NCT03666559 and NCT03922555). Overall, predicted single drugs implicated in oxidative stress and polyamine metabolism pathways are more target-specific than AntiBCs’ transcription factors, of which fotemustine, arsenic trioxide and valganciclovir represent repurposable single drugs for glioma with a clinical profile comparable to AntiBCs.

We further searched for drugs that could increase the potency of AntiBCs and IAGs to predict drug combinations that allow targeting pathways characterized by many isozymes and alternative reactions that must be inhibited simultaneously, avoiding pathway redundancy of the AntiBCs combinations. The predicted drug combinations allowed targeting CA, GSH exchange, glutamate, polyamine and GSH biosynthesis. In addition to inhibiting ABC transporters, anti-hypoxic AntiBCs reduce angiogenesis through mTOR/HIF-1α/VEGF or RAF/MEK pathways, while anti-hypoxic combination drugs target their downstream CA and MAO-A/B genes. The relatively high potency and xenograft growth reduction of anti-hypoxic AntiBCs compared to alkylating AntiBCs is clinically consistent with the superior median overall response rate of anti-angiogenic agents compared to alkylating agents (6.1%) in rGBM phase II trials [72]. CA2, CA9 and CA12 are highly expressed in GBM, especially CA9, which is not expressed in a healthy brain [73] and is significantly correlated with poor survival in GBM [73] and the AST grade [74]. The proposed 17 drug combinations include pairs of a total of 19 drugs. In brain cancer, 18 out of 19 combinations drugs (except rifamycin) have been tested individually. However, none of the 17 proposed combinations has been assessed *in vivo* or *in vitro* for brain cancer, which warrants their testing. Even though three combinations had synergistic effects *in vitro* in non-brain cancer, two failed in clinical trials. Among the 17 predicted combinations, three IAGs: cannabidiol, eflornithine and fluorouracil, were predicted to have a synergistic effect with adapalene (GOT1-inhibitor), rifamycin (SLCO-inhibitor) and zonisamide (CAi), respectively. With cannabidiol and rifamycin being ABC transporter inhibitors, cannabidiol/adapalene and eflornithine/rifamycin combinations present potential combinations for relapsed glioma from AntiBCs resistance. Additionally, iGBM accurately predicted ABCG2 transporter responsible for efflux activity against protoporphyrin, as GBM-specific vulnerability, matching the impaired heme biosynthesis in IDH-mutant glioma [29]. Among the 19 combination drugs, zonisamide (selective CA9-inhibitor, approved for seizures) had the highest potency and CSF bioavailability and target additionally MAOA/B increasing its anti-hypoxic action like procarbazine. While adapalene has poor CSF bioavailability, the recent nanoparticle formulation of adapalene increased its CSF bioavailability [75]. Similarly, the lack of survival of eflornithine monotherapy in GBM but not AST in a phase II clinical trial [49] highlights the importance of testing the eflornithine/rifamycin combination in GBM.

Some of the shared genes between Recon3D reconstruction and DepMap’s common essential genes are enriched for other functions unrelated to growth (Table S17). As the formulation of the biomass was shown to impact essential gene predictions [53], a tailored biomass formulation should improve accuracy. Besides curating the biomass growth formulation and updating GPR rules, disease- specific reactions could be added before model building to improve essentiality analysis and drug prediction [53]. For example, adding IDH-mutant biochemical reactions would allow more accurate modeling of the LGG. Similarly, formulating evaluation tests of predicted essential genes on the DepMap scores would allow reproducible predictions and evaluations between studies. Moreover, Flux Variability Analysis of metabolic exchanges could be improved using random sampling, especially for reactions with a wide flux range [76]. While the iCTRL model was built from four healthy brain samples from TCGA-GBM, building a healthy brain model from the GTEx expression data [77] with CSF medium or using the brain model from the whole-body model [13] could advance evaluating the safety of the predicted drugs. In summary, metabolic modeling predicted combinations that overcome the target redundancy of the AntiBCs combinations with alternative, accurate glioma subtype–specific targets. The top two combinations present a safety profile and ABC substrate inhibition for one of the two drugs, with variations in subtype specificity that call for further testing.

## CONCLUSION

GBM and LGG suffer from poor patient survival and accurate preclinical models, respectively, that hinder new therapies’ discovery. Moreover, AntiBCs lack either adequate potency or CSF bioavailability. In addition, two-thirds of AntiBCs are ABC transporter substrates, increasing their drug resistance and core tumor diffusion. In this work, we present glioma subtype–specific GEMs to predict single drugs and combinations that are promising candidates to be translated into clinical trials. Among others, LGG GEMs accurately predicted glutamate and GSH biosynthesis vulnerabilities, while GBM GEM accurately predicted glutamine dependency and heme biosynthesis. Unlike the target redundancy of the combination AntiBCs, predicted combinations target alternative reactions, potentiating their synergism. Of the predicted 33 single drugs (19 combinations drugs), half were effective *in vitro,* and 17 [8] were tested against GBM PDXs, of which 11 [5] were effective. Similarly, predicted drugs show comparable or improved CSF bioavailability to AntiBCs. Despite five cell cycle inhibitors failing in phase II glioma clinical trials due to conceivably being ABC transporter substrates, two single drugs (fotemustine and valganciclovir) and two combination drugs (eflornithine and celecoxib) exceeded the primary survival outcome alone or combined with AntiBCs in phase I/II clinical trials. Our work warrants fotemustine as pan-glioma monotherapy and eflornithine/rifamycin and cannabidiol/adapalene as promising new combinations for GBM and LGG, respectively.

**Key Points**

- Glioma metabolic modeling accurately captured inter-subtype metabolic variations.
- Fotemustine showed very good performance in a meta-analysis in OS.
- Fotemustine alone and eflornithine in combination showed improved survival in glioma trials.
- The prediction of glutamate depletion by adapalene in IDH-mutant glioma agrees with literature.

## Supplementary Material

Supplementary_File_1_bbae199

Supplementary_File_2_bbae199

## Data Availability

All analyzed data are publicly available from the Resources Table in Supplementary Table 1. Analysis code is available under https://github.com/sysbiolux/GliomaGEM/

## Author Biographies

**Ali Kishk** is a PhD graduate from the University of Luxembourg in Systems Biology. His research focuses on drug repurposing for cancer using metabolic modeling.

**Maria Pires Pacheco** specialises in metabolic modelling and especially the development of workflows for the integration of omics data in metabolic models.

**Tony Heurtaux** is Research Scientist at the Department of Life Sciences and Medicine (University of Luxembourg) and the Luxembourg Centre of Neuropathology. His research work is focused on molecular and cellular biology of glial cells.

**Thomas Sauter** is full professor at the Department of Life Sciences and Medicine, University of Luxembourg. His research is focused on computational systems biology.
