# Supplementary material for: Metabolic models predict fotemustine and the combination of eflornithine/rifamycin and adapalene/cannabidiol for the treatment of gliomas
Source: Brief Bioinform. 2024 May 2;25(3):bbae199. doi: 10.1093/bib/bbae199 (PMC11066901; doi:10.1093/bib/bbae199)
Supplement: Supplementary_File_1_bbae199 [file supplementary_file_1_bbae199.docx]

**Running title: Prediction of repurposable drugs and drug combinations for glioma**

*Metabolic models predict fotemustine and the combination of eflornithine/rifamycin and adapalene/cannabidiol for the treatment of gliomas*

Ali Kishk^1^, Maria Pires Pacheco^1^, Tony Heurtaux^1,2^, Thomas Sauter^1*^

1. University of Luxembourg, Department of Life Sciences and Medicine, L-4367 Belvaux, Luxembourg
2. Luxembourg Center of Neuropathology, L-3555 Dudelange, Luxembourg

* Corresponding author: [Thomas.Sauter@uni.lu](mailto:Thomas.sauter@uni.lu)

**Supplementary Methods:**

To repurpose FDA-approved drugs for the glioma subtypes, building high-quality semi-curated GEMs is crucial. Subtype intra-homogeneity and separability between subtype samples were critical factors assessed before the subtype model building.

1. **Sample stratification based on** **WHO 2021 glioma subtypes**

The 2021 World Health Organization (WHO) classification of the central nervous system (CNS) tumors ^1^ defined two key molecular markers for glioma subtype classification: IDH mutation status, 1p.19q codeletion. Two RNA-Seq datasets in The Cancer Genome Atlas (TCGA) were selected for model building: TCGA-GBM for glioblastoma (GBM) and TCGA-LGG for lower-grade glioma (LGG)^2^. The metadata for both datasets was downloaded using the R package *TCGABiolinks*^3^ containing three metadata features (IDH mutation status, 1p.19q codeletion, and diagnosis). Both molecular features were used for sample selection and stratification (**Figure S1**). Based on the 2021 WHO classification of CNS tumours^1^, IDH-mutant GBM has been moved into IDH-mutant astrocytoma (AST). To ensure intra-homogeneity between subtype samples and constancy to the 2021 WHO classification, IDH-mutant GBM samples were discarded. Moreover, samples with oligoastrocytoma diagnosis ("mixed-glioma") were excluded as they were poorly defined and represented a mixture of AST and oligodendroglioma (ODG) in different proportions between patients. The selected samples for the three glioma subtypes were classified as follows:

- iGBM: diagnosis = glioblastoma and IDH mutation = wildtype.
- iAST: diagnosis = astrocytoma, IDH mutation = mutant and 1p.19q codeletion = no codeletion.
- iODG: diagnosis = oligodendroglioma, IDH mutation = mutant and 1p.19q codeletion = codeletion.


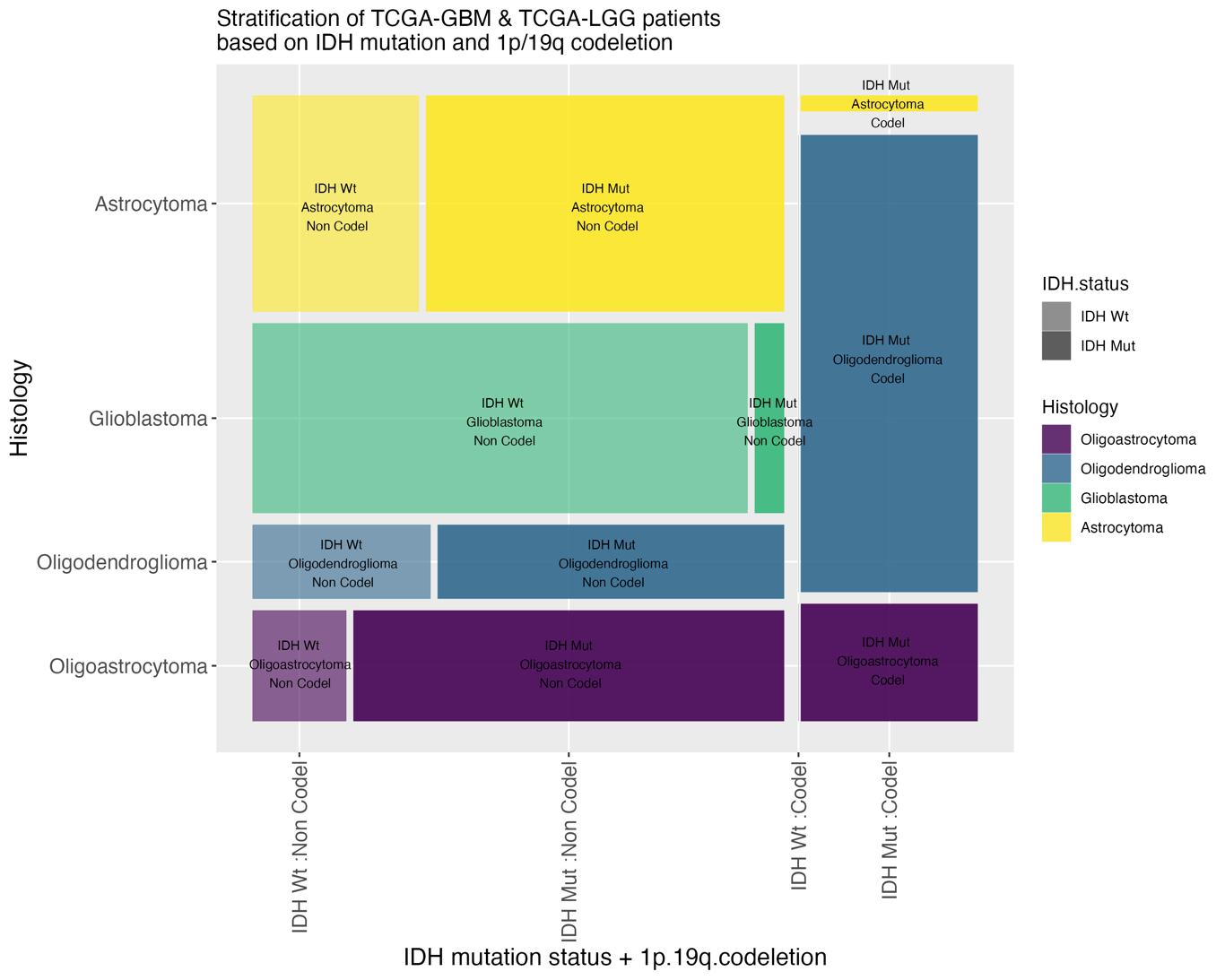


**Figure S1: TCGA metadata stratification based on 2021 WHO classification of CNS tumors using diagnosis, IDH mutation status, and 1p.19q co-deletion.**

1. **Model selection (choosing the optimal combination of preprocessed data, generic model, and curation)**

Many factors may affect the contextualization and specificity of the subtype models, such as the choice of generic models, applied curation, the objective function(s), and preprocessed data. Therefore, several settings were tested to find the optimal combination of preprocessed data, generic model, and medium that ensured the best separation between the sample models. The search included three preprocessed data, generic metabolic models (Recon2^4^, Recon3D^5^ or Human1^6^), and two curation methods (no medium or with cerebrospinal fluid medium (CSF)). The three preprocessed data are Rahman2015^7^, Ceccarelli2016^8^ and TCGABiolinks^9^. These datasets were downloaded in FPKM (Fragments Per Kilobase of transcript per Million mapped reads) format. The CSF medium composition was imported from the Human Metabolome Database^10^ like the whole-body metabolic model^11^.

**2.1. Separation between the glioma subtypes’ sample models**

These three factors were used for sample model building using rFASTCORMICS (Pacheco et al., 2019). rFASTCORMICS is a context-specific metabolic model-building algorithm that takes as inputs RNA-Seq data and a consistent generic model^12^ with an optional medium composition. The default discretization function in rFASTCORMICS (*discretize_FPKM*) was used for Ceccarelli2016 and Rahman2015 as their expression distribution peaked at the rightmost side with unexpressed formed a leftmost tail (See **Figure S2**). Meanwhile, TCGABiolinks was discretized using another function (*discretize_FPKM_skewed)* as the expression data had a bimodal distribution with two peaks with comparable heights. In total, 18 combinations were searched, and 358 samples were built for each combination. Biomass production and ATP production were chosen as objective functions. The median number of samples' reactions was computed for each combination, and a hierarchical clustering of the samples was plotted as a metric for separating the three subtypes. After selecting the optimal combination of data, generic model, and curation, three subtype models were built using the abovementioned settings.

**2.2. Evaluation to literature-retrieved exchange reactions**

In addition to sample model separation, subtype models of the various data-model-curation combinations were evaluated against literature-retrieved metabolic exchanges that are representative of the within subtype variations (**Table S2**). Flux Variability Analysis (FVA) was applied for the various subtype models exchanges with 100% maximization of the biomass objective function and compared to the literature-retrieved metabolic. Rahman2015 data, Recon3D generic model, and CSF curation showed the best matching to literature-retrieved metabolic exchanges (**Figure S5**). Similarly, the same setting showed the best sample model separation (**Figure S3**), suggesting that subtype separation and empirical metabolic exchange matching could improve model selection.

**2.3. Evaluation to cancer common essential genes**

To further evaluate if other input reconstruction and prepossessing methods would impact the essential genes prediction. We ran the single gene deletion workflow on all possible data-model-curation combinations and compared the respective essential genes to the list of common genes (defined as genes found to be essential in >90% of cell lines in pan-cancer CRISPR-Cas9 screens) by the Cancer Dependency Map’s (DepMap)^13^. All models had accuracy between 0.84-0.90, with Recon3D with and without medium constraints, as well as Human1 having similar accuracy values (above 0.88). However, without medium constraints, Recon2 and Recon3D did predict very few essential genes also captured by the constrained conditions and Human1 is slightly advantage by the larger number of genes included in the models. Human1 has 3050 genes against 1884 and 1733 for Recon3D and Recon2, inflating so the number of True Negatives. Finally, as we are comparing our predictions to common essential genes (no good quality glioma-specific binary high throughput screens are available), some predicted essential genes corresponding to glioma-specific essential genes might be wrongly identified as False Positives (**Figure S6**).


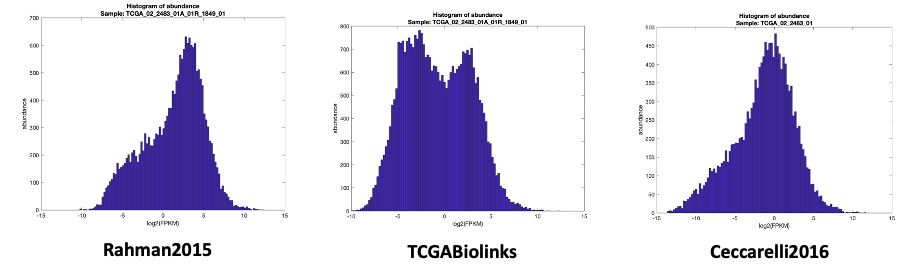


**Figure S2: Different TCGA RNA-seq data showed different distributions affecting the choice of discretization during model building.**

**Table S1: CSF medium constraining reduced sample model sizes by >20% in Recon2 and > 25% in Recon3D.** Sample models were built to determine the optimal combinations of data, generic model, and medium for model separation. The median number of reactions for sample models was computed for each parameter combination. Sample models built with Human1 with CSF medium were missing the objective function (OF) and thus were excluded. The total number of reactions in each generic model is displayed beside the generic model names. Noticeable reduction in model sizes with CSF medium, especially in Recon3D, indicated a separation using a biologically representative medium constraining.

| **Data** | **Medium** | **Generic model** | | |
| --- | --- | --- | --- | --- |
|  |  | **Recon2 (5317)** | **Recon3D (10600)** | **Human1 (11887)** |
| TCGABiolinks | No medium | 2752 | 5388 | 6533 |
| Ceccarelli2016 | No medium | 2999 | 5465 | 6297 |
| Rahman2015 | No medium | 2701 | 5183 | 6279 |
| TCGABiolinks | CSF medium | 2310 | 3862 | Missing OF |
| Ceccarelli2016 | CSF medium | 2295 | 3732 | Missing OF |
| Rahman2015 | CSF medium | 2258 | **3776** | Missing OF |


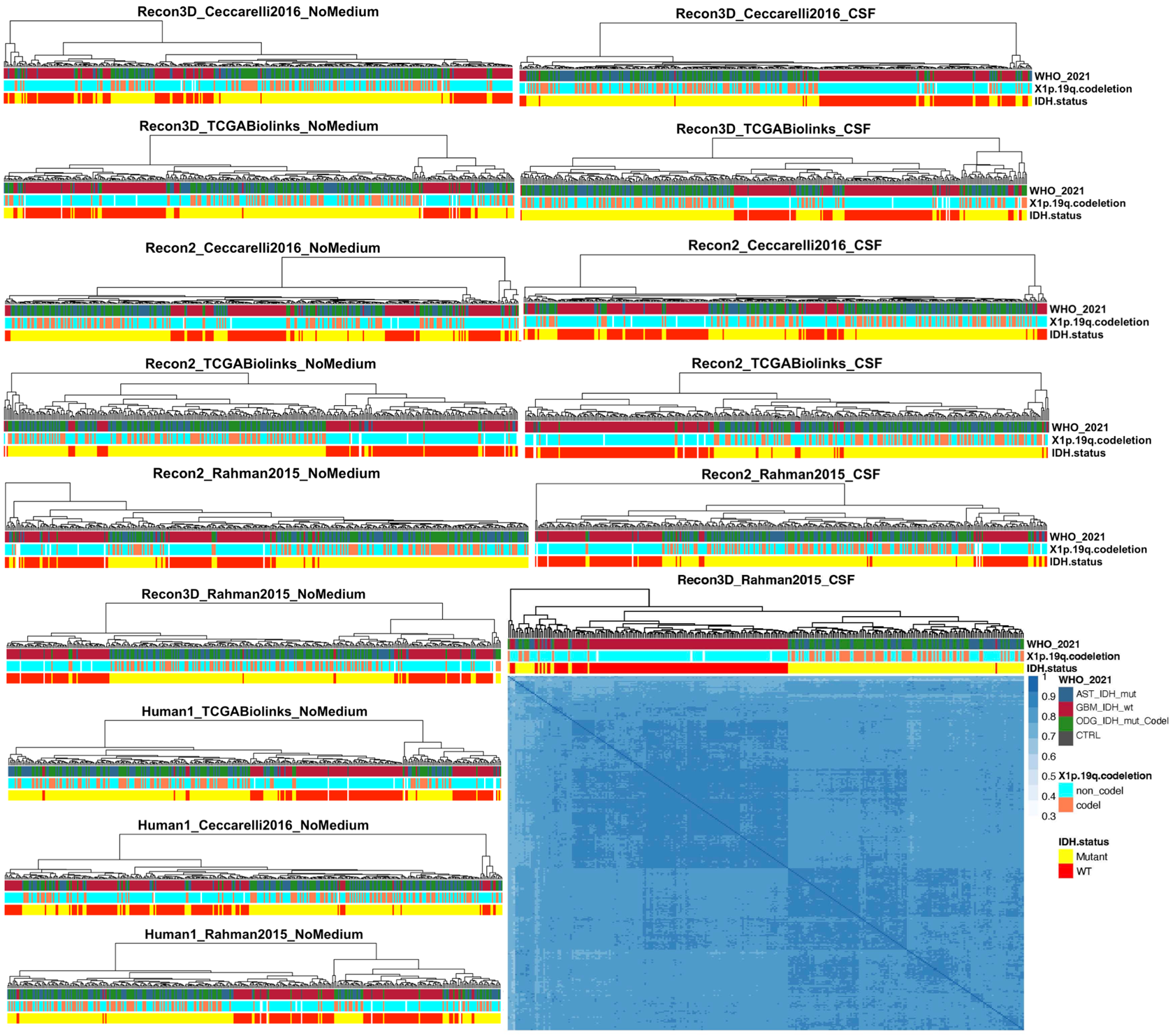
**Figure S3: The optimal setting using Rahman2015 data, Recon3D generic reconstruction, and CSF curation improved the separation between the sample models.**
Hierarchical clustering was applied for the sample models' reactions of each setting separately to define the best setting in intra- and inter-heterogeneity of the three subtypes. CSF medium constraining showed enhanced separation, but none separated AST and ODG. Only Rahman2015 data, Recon3D generic model, and CSF curation allowed a separation between GBM and LGG (AST and ODG).


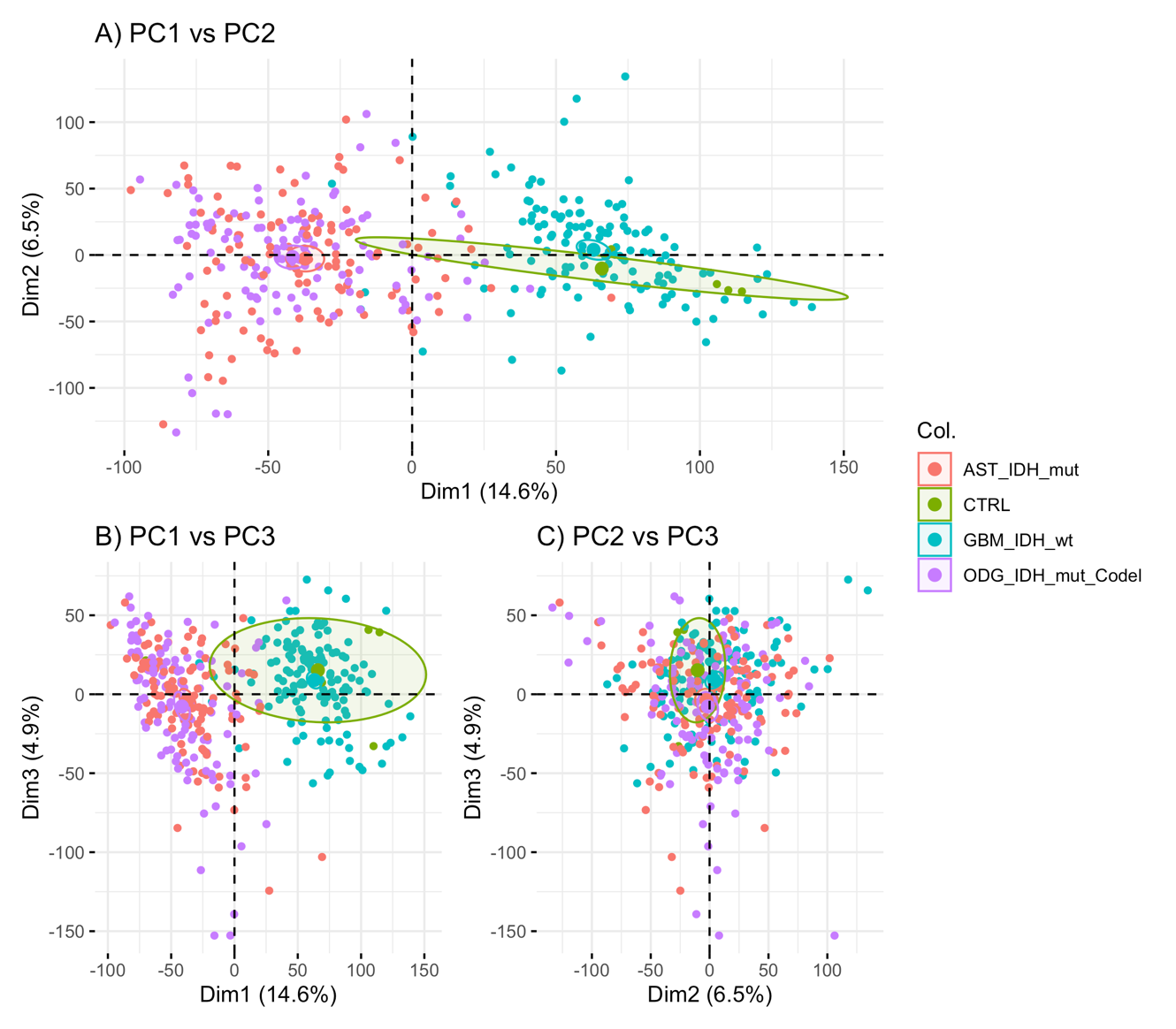


**Figure S4: Principal component analysis of the TCGA data expression data of Rahman2015 shows a clear separation between GBM and LGG but not between AST and ODG.**

To evaluate the quality of the TCGA expression data, principal component analysis **(**PCA) was used to examine the separation between the three glioma subtypes based on the 2021 WHO CNS tumors classification. Separation based on IDH mutation was clear but not based on 1a/19q co-deletion, suggesting the closeness between AST and ODG.


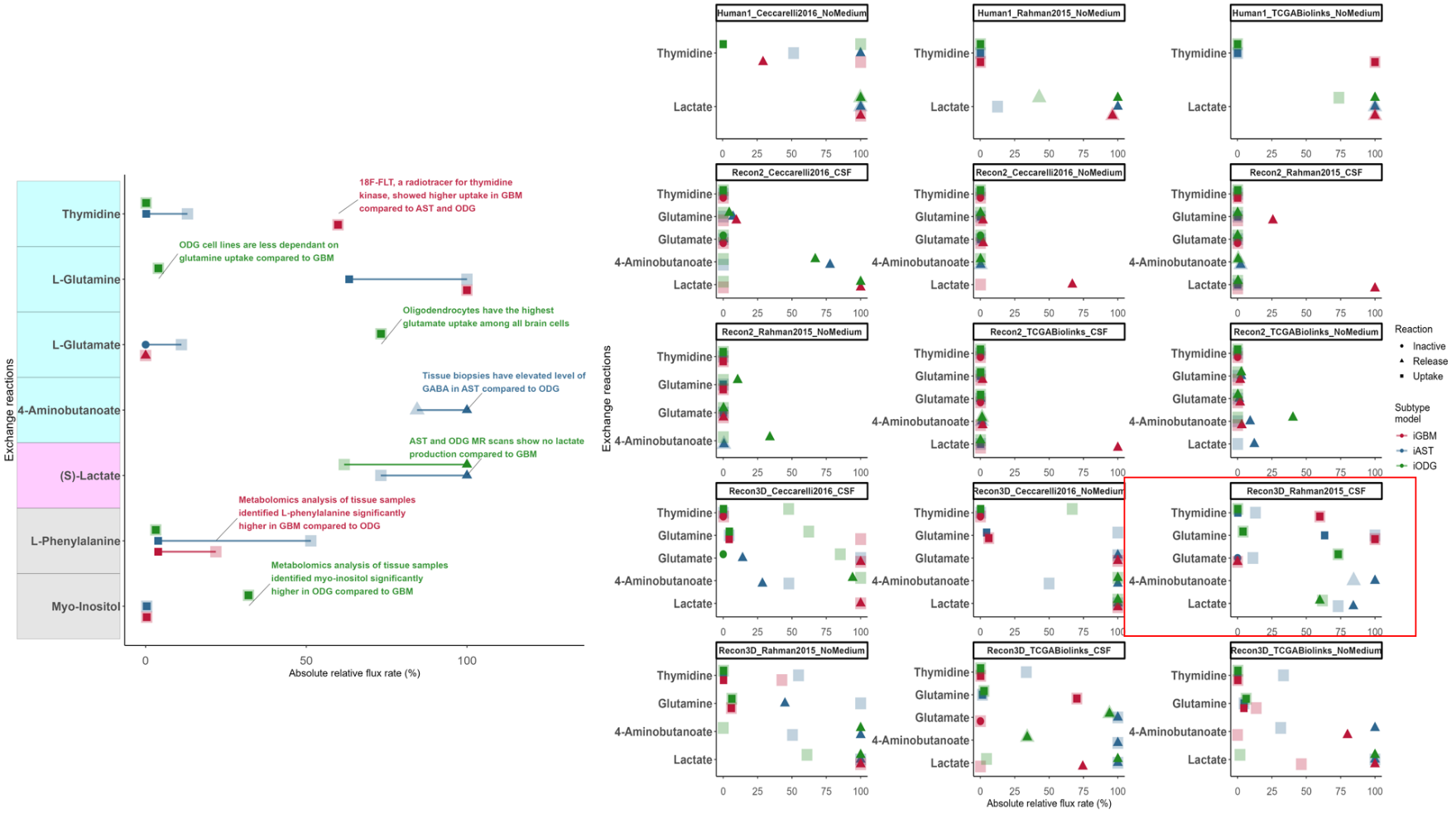


**Figure S5: Flux variability analysis determined the optimal setting using Rahman2015 data, Recon3D generic model, and CSF curation to better match literature-retrieved metabolic exchanges.**Flux variability analysis was applied to the various subtype model settings to determine which is the best setting matching literature-retrieved subtype-specific metabolic exchanges (**Table S2**).


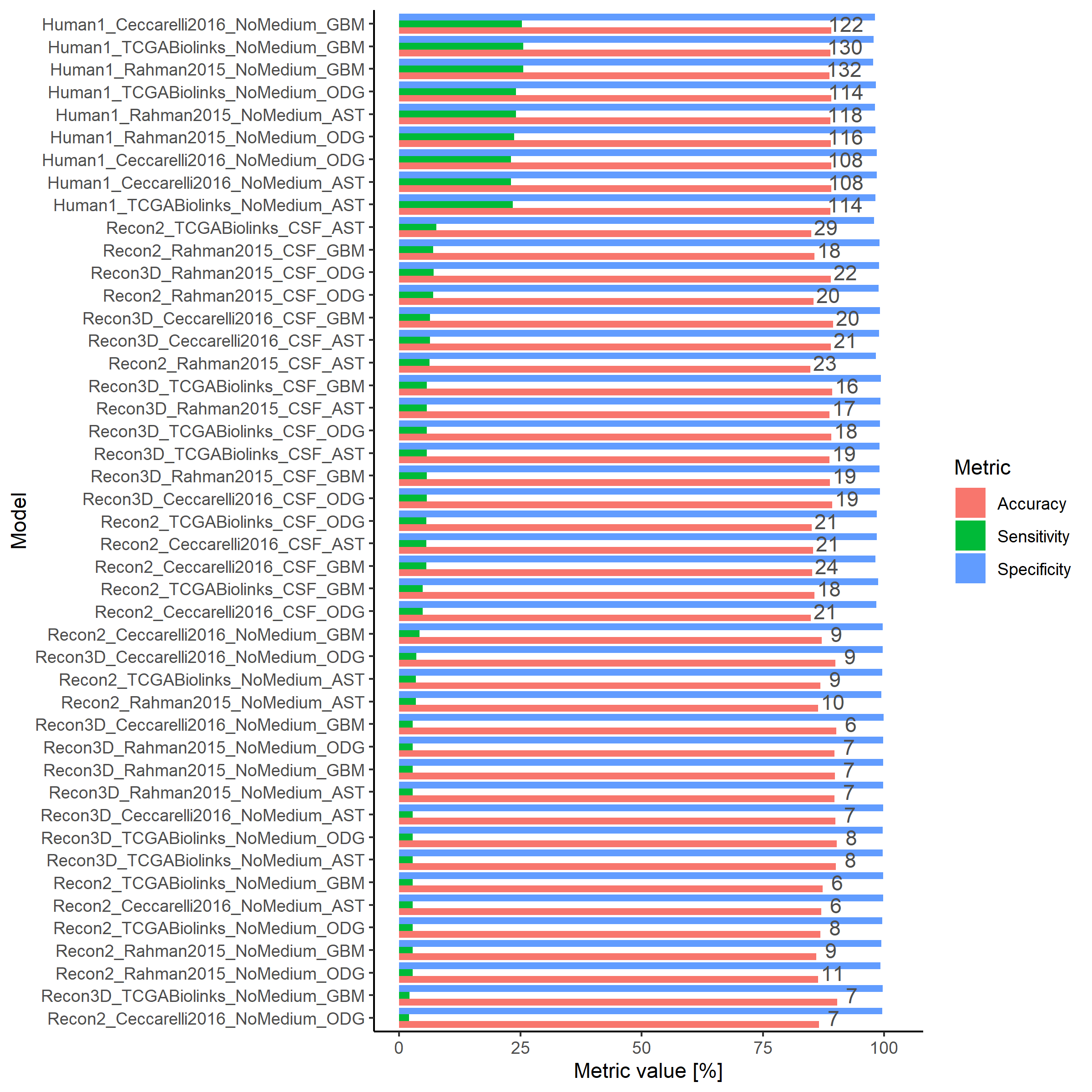


**Figure S6: The accuracy for all models lies between 0.84 and 0.9 *for* capturing common essential genes.**

Recon2 and Recon3 models without medium constraint have a lower number of predicted essential genes. Human1 is advantaged by the larger number of metabolic genes included in the model which inflates the number of True Negatives.

**3. Drug prioritization**

Different clinical, preclinical, and pharmacokinetics data in brain cancer were collected to rank and filter the predicted single drugs and combinations based on their eligibility for testing (**Supplementary File 2, Tables S8-10**). The data collected in **Supplementary File 2** included clinical trial data (**Supplementary File 2, Table S11**), *in/ex vivo* xenograft testing (**Table S12**), *in vitro* potency (**Supplementary File 2, Table S13**), non-brain cancer cytotoxicity (**Supplementary File 2, Table S14**), blood-brain barrier (BBB) permeability, CSF bioavailability (**Supplementary File 2, Table S15**), and possible drug-drug interactions for combinations (**Supplementary File 2, Table S16**). Other selection criteria included diversity and main mode of action (MOA), proven efficacy in higher clinical evidence, and agreement of predicted MOA with literature. Single drugs that showed either *in vitro* increased proliferation, no effect, or substrate drug-target interaction were excluded. Six of the ten excluded single drugs were hormones/co-factors, followed by two antivirals and two psychoactive drugs. The remaining drugs were ranked into selected candidates and weak candidates (see **Supplementary File 2, Table S8**). The final benchmarking assessed the predicted drugs compared to the approved anti-brain chemotherapies (AntiBCs) using the criteria summarized in **Table S6.** The final evaluation classified the predicted single and combination drugs into effective, ineffective, and untested drugs across *in vitro*, *in/ex vivo* xenografts, and phase II clinical trials.

**3.1. Clinical trial data**

Predicted single and combination drugs were searched on the ClinicalTrials website ([beta.clinicaltrials.gov](http://beta.clinicaltrials.gov/)) using ("Brain cancer" OR "Glioma") as the condition. Further clinical trials retrieved by using drug synonyms from Google Scholar were added. Due to the absence of a systematic database of the clinical trials, trials clinical trial identifiers were searched in PubMed to find which had published results. Among the clinical trials with published results, two main survival measures were shared as the primary outcome: overall survival (OS) and progression-free survival (PFS, duration between treatment and symptom worsening). If available, both survival measures were collected for phase I/II or higher clinical trials. The clinical trial data covered 50 phase I/II or higher brain cancers (**Supplementary File 2, Table S11**), of which seven are two-arm glioma trials where an arm is an approved treatment.

**3.2 Potency and pharmacokinetics data**

*In vitro* data for the predicted single and combination drugs were retrieved from high-throughput drug screening (see **Table S5**) and literature (**Supplementary File 2, Table S13**). The in *vitro* data in the literature ranged from increased proliferation, no effect, minimum inhibitory concentration, IC_50_, viability reduction, and apoptosis/autophagy. In literature, if a drug lacks *in vitro* data in brain cancer cell lines, data were collected in non-brain cancer cell lines (**Supplementary File 2, Table S14**). *In vitro* data from high-throughput drug screening covered only two measure types (viability reduction and IC_50_). Pharmacokinetics data (CSF bioavailability, BBB permeability, and ABC (ATP Binding Cassette) transporter affinity) were collected from B3DB^14^, NCATS Inxight Drugs^15^, and the literature (**Supplementary File 2, Table S15**).


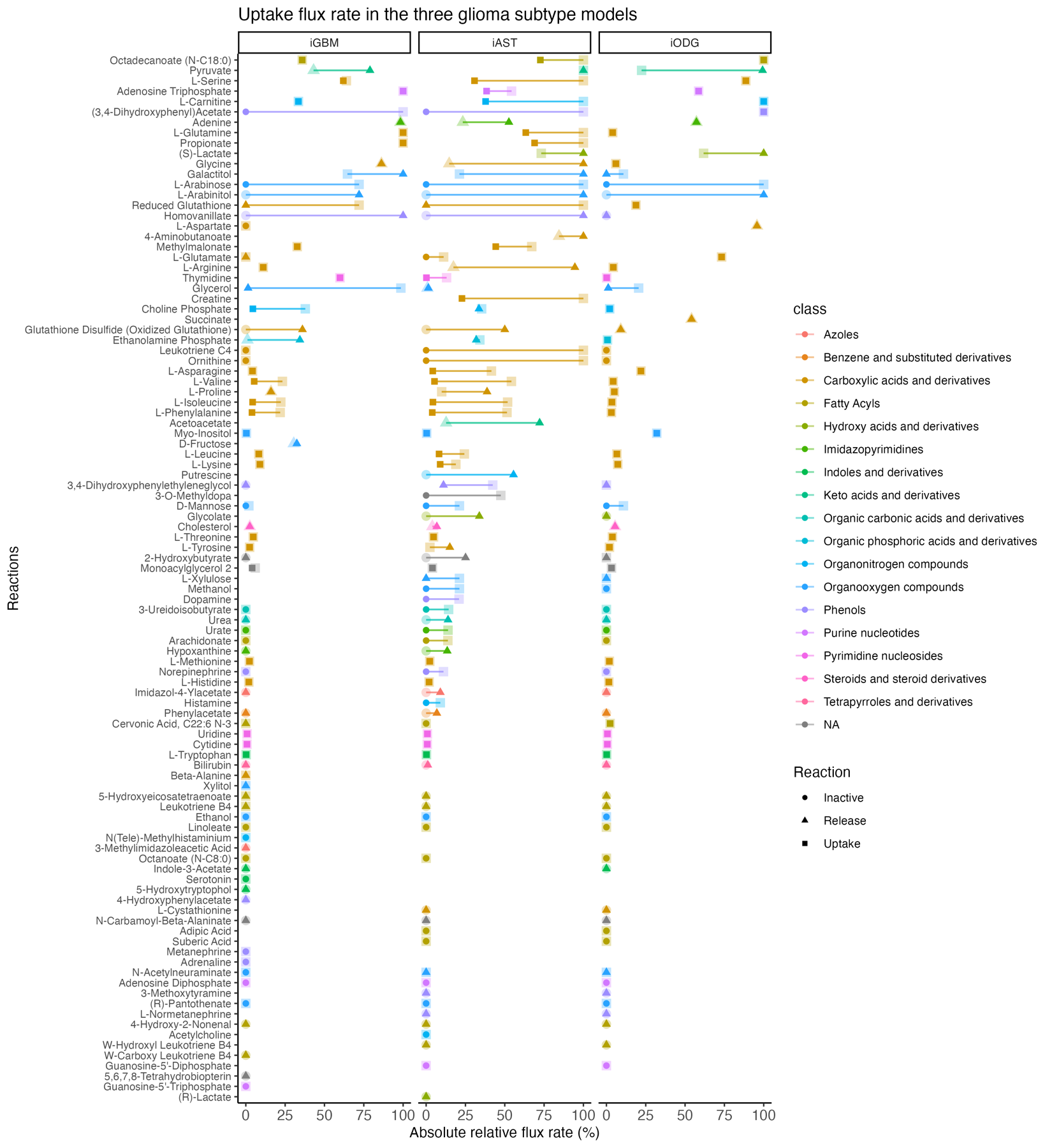
**Figure S7: Flux variability analysis of the metabolite exchanges identified differential uptake and production between the glioma subtypes.**

As a quality control, the preferred metabolite exchanges were compared to known variations in the literature. The 101 metabolite exchanges shared across the three glioma subtype models were fed to the *fluxVariability* function from the COBRA Toolbox v3.0 to define reactions with narrow fluxes predicted to influence biomass growth.

**Table S2: Predicted exchange reactions showed that six out of seven were consistent with previous knowledge.**

| **Reaction** | **Prediction** | **Literature evidence** | **Literature related subtype** | **Matching with literature** | **Cell Lines/ Tissue samples** | **Reference** |
| --- | --- | --- | --- | --- | --- | --- |
| Thymidine uptake | Thymidine uptake has a narrow boundary in **GBM** alone, with a small influx in **AST** and **ODG** | 18F-FLT, a radiotracer for thymidine kinase, showed higher uptake in GBM compared to AST and ODG | GBM | Yes | Patient scans | ^16,17^ |
| L-Glutamine uptake | Glutamine uptake has a narrow boundary, and it was higher in **GBM,** followed by **AST** with a minute flux in **ODG** | ODG cell lines are less dependent on glutamine uptake compared to GBM | ODG | Yes | ODG: Hs683; HOG | ^18^ |
| L-Phenylalanine uptake | L-phenylalanine flux uptake has a broader range in **GBM** than in **ODG** | Metabolomics analysis of tissue samples identified L-phenylalanine as significantly higher in GBM compared to ODG | GBM | Yes | Tissue samples | ^19^ |
| Myo-Inositol uptake | Higher myo-inositol uptake was observed in ODG compared to AST and GBM | Metabolomics analysis of tissue samples identified myo-inositol as significantly higher in ODG compared to GBM | ODG | Yes | Tissue samples | ^15^ |
| 4-Aminobutanoate release | 4-Aminobutanoate released only in **AST** | Tissue biopsies have elevated levels of GABA in AST compared to ODG | AST | Yes | Tissue samples | ^20^ |
| L-Glutamate uptake | Glutamate uptake was predicted to be active in ODG but not in AST or GBM | Oligodendrocytes have the highest glutamate uptake among all brain cells | ODG | Yes | Human fetal oligodendrocytes | ^21^ |
| (S)-Lactate release | Lactate exchange was predicted to be reversible in AST and ODG but inactive in GBM | AST and ODG MR scans show no lactate production compared to GBM | ODG | No | Patient scans | ^22^ |

***
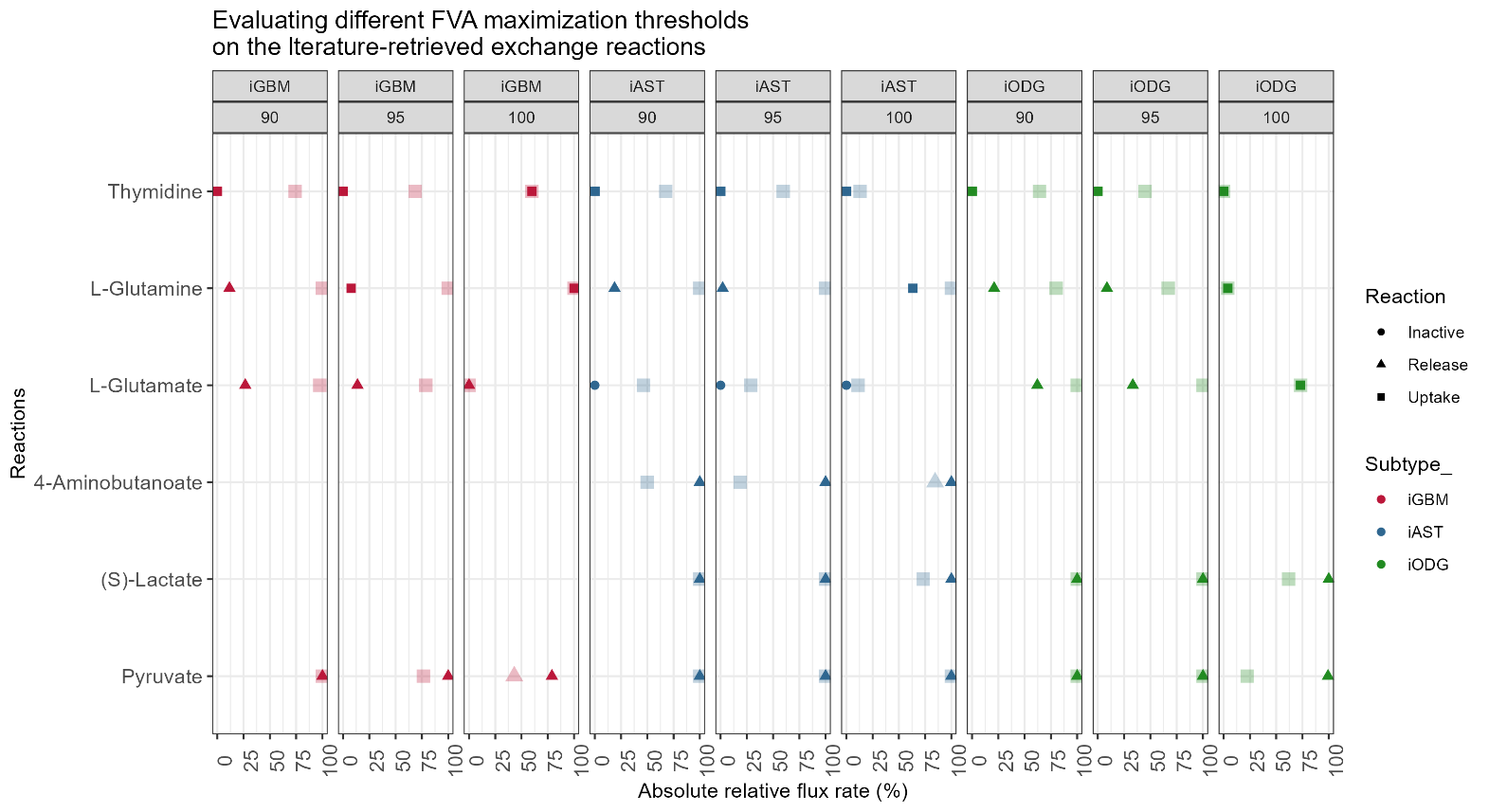
***

**Figure S8: A release of the optimization threshold from 100% to 95% and 90% increases the range predicted flux drastically turned some exchange reactions with narrow bounds at 100% to become unbounded.**

To determine the optimal maximization threshold for the Flux Variability Analysis (FVA) matching the literature-retrieved exchange reactions (**Table S2**), different thresholds (90%, 95%, and 100%) were tested for the three glioma subtype models. Increasing the maximization threshold for the biomass reaction, decreased the range of the metabolic flux and improving their matching to the literature. For example, the known glutamine dependency in GBM and glutamate dependency in ODG were predicted with 100% maximization.

**Table S3: A literature search identified six of the 25 predicted essential genes linked to glioma viability, resistance, or patient survival.**

| **Gene** | **Function** | **Literature support for glioma** | **Cell lines/ Patient samples** | **Reference** |
| --- | --- | --- | --- | --- |
| TXNRD1 | Thioredoxin reductase | •Selective inhibitors of TXNRD1 induced cell death and decreased proliferation, invasion, and migration of multidrug-resistant glioma cell lines  •Selective inhibitors of TXNRD1 showed synergistic effects with temozolomide. | U87, U87-TxR, C6, RC6 (Rat) | ^23^ |
|  |  | Selective inhibition of thioredoxin reductase was more cytotoxic than TMZ treatment. | GL261 | ^24^ |
|  |  | • TXNRD1 overexpression linked to radio-resistance • KD of TXNRD1 reduced radio-resistance in GBM. | U87MG, T98G | ^25^ |
|  |  | • Upregulated in > 66% of cases.  •Significantly linked to higher proliferation and poorer prognosis | AST patients | ^26^ |
|  |  | • Linked to poor diagnosis and higher grade | ODG patients | ^27^ |
| SLC27A4 | Fatty acid uptake | SLC27A3 knockdown, SLC27A4 isoform, reduced GBM xenograft growth, and its expression was linked to GBM stemness | Human GBM neurosphere lines | ^28^ |
| RRM2 | Nucleotide interconversion | Genetic knockdown of RRM2 sensitizes GBM cell lines to TMZ in vitro and in vivo | U87 | ^29^ |
| RRM1 | Nucleotide interconversion | High-throughput drug screening followed by shRNA of the hit's targets identified RRM1 among nine genes as GBM vulnerabilities | U87, U87vIII, T98G, GL261 | ^30^ |
| SPTLC1 | Sphingolipid synthesis | Knocking down SPLTC genes reduced the viability of GBM cell lines | U87MG | ^31^ |
| ANPEP | Glutathione metabolism | ANPEP is highly expressed in GBM and linked to low survival | ANPEP is highly expressed in GBM and linked to low survival | ^32^ |
| SLC6A14 | L-Arginine uptake | Arginine deprivation reduced cell adhesion and invasiveness of GBM cell lines | U251MG, U87MG | ^33^ |


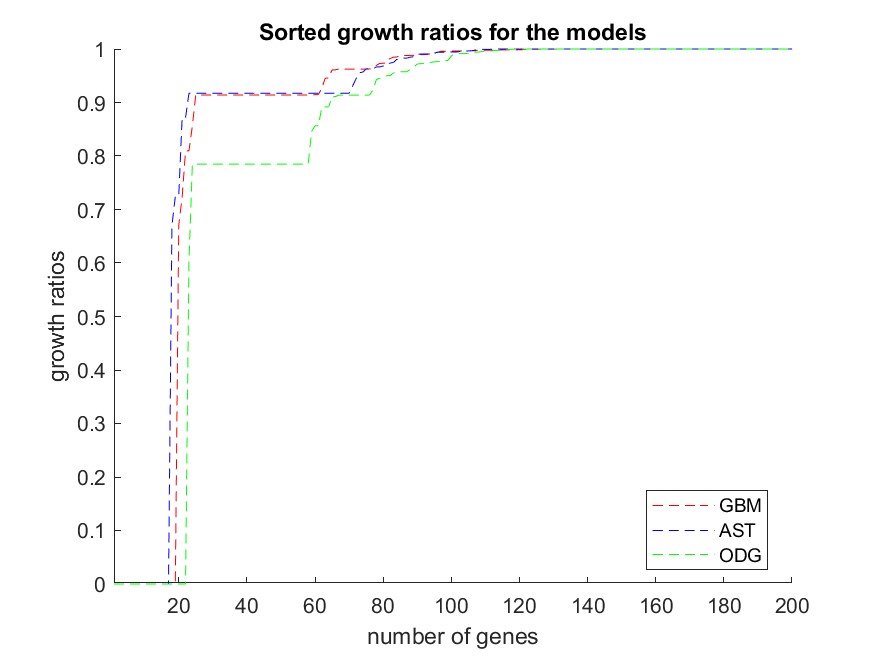


**Figure S9: The number of predicted essential genes remained constant for growth ratio between 0 and 0.7.** Above 0.7, the number of essential genes substantially increased but the growth reduction is too modest to consider using these additional genes as cancer targets.


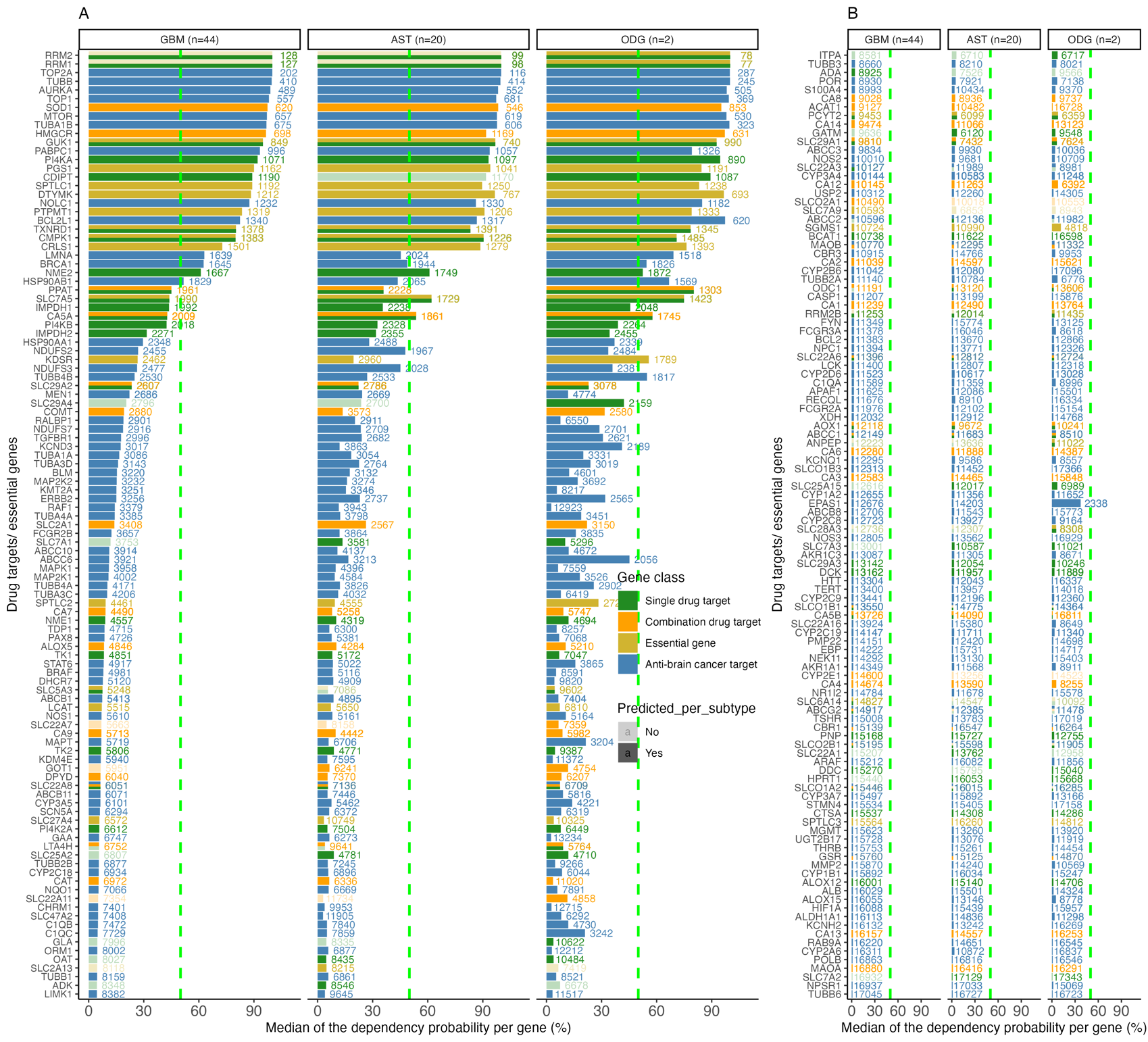


**Figure S10: Only RRM1 and RRM2 of the predicted essential genes involved in nucleotide biosynthesis showed stronger gene dependency than AntiBCs targets.**

To evaluate predicted drugs against AntiBCs on target essentiality in glioma cell lines, targets of AntiBCs, predicted single and combination drugs and essential genes were ranked according to the dependency probability in the DepMap database. DepMap is the largest database of genome-scale *in vitro* CRISPR-Cas9 KO screening of cancer cell lines, with dependency probability representing the likelihood of cell death upon gene KO. Genes were ranked by median dependency probability in GBM cell lines, and genes not predicted in a subtype were highlighted in transparent color. RRM1 and RMM2 targeted by anti-metabolites exceeded AntiBCs targets, followed by non-alkylating AntiBCs targets (doxorubicin (TOP2A, AURKA, TOP1) and vincristine (TUBB, TUBA1B)).

**Table S4: Five predicted drugs matched in the targeted genes and reactions for their anti-glioma activity in the literature.**

| **Drug** | **Evidence from Literature** | **Glioma cell lines** | **Predicted targets** | **Predicted Reactions** | **Reference** |
| --- | --- | --- | --- | --- | --- |
| Arsenic-trioxide | Arsenic-trioxide induces cytotoxicity by increasing ROS formation. | C6 and 9L | TXNRD1 | Ph[c] + nadph[c] + trdox[c] -> nadp[c] + trdrd[c] | ^34^ |
| Acetazolamide and brinzolamide | Both drugs are chemosensitizer for TMZ by reducing extracellular acidosis. | U373, U251, U87MG, GaMG, U87 and GSCs | Carbonic anhydrases | H20 + CO^2^ <=> H^+^ + Bicarbonate | ^35^ |
| Cannabidiol | Increasing ROS and depletion of glutathione. | U87 | ACAT1, CAT, GSR | •2 H_2_O_2_-> 2 Water + Oxygen  •gthox[c] + h[c] + nadph[c] -> 2.0 gthrd[c] + nadp[c] | ^36^ |
| Eflornithine | Eflornithine reduces GBM cell viability by targeting ODC1 | DIPG | ODC1 | L-glutamate biosynthesis from 2-oxoglutarate | ^37^ |

**.
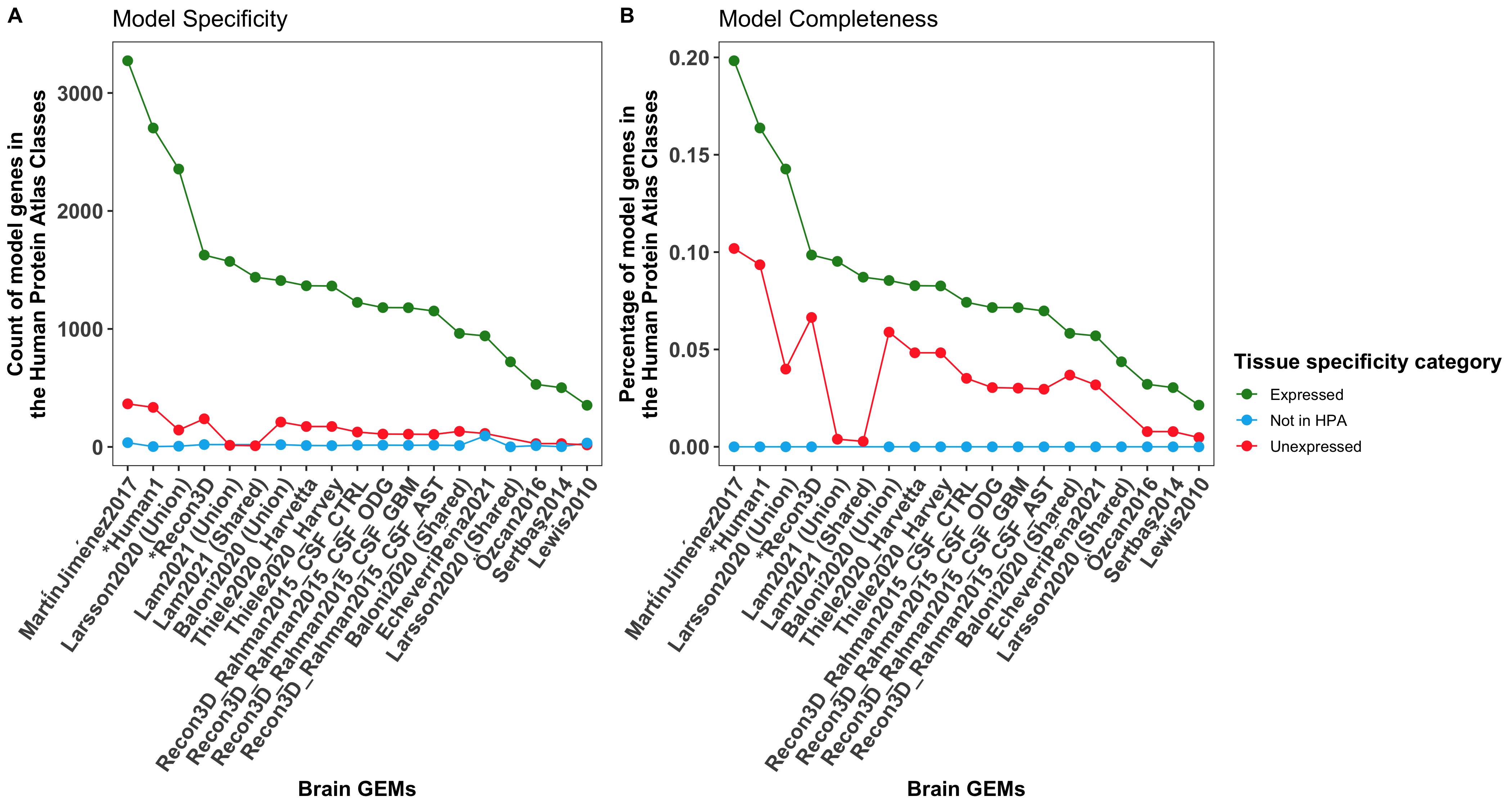
**

**Figure S11: Glioma subtype models’ genes showed comparable specificity and completeness to curated and semi-curated brain metabolic models according to** **the Human Protein Atlas brain-specific category.**

The genes of the glioma subtype models were compared to the ones included in the brain metabolic models discussed in our previous review^38^ using the Human Protein Atlas^39^ (HPA) as reference to determine the quality of each model. HPA gene categories classify genes based on differential tissue expression of the brain, that were combined into “Expressed” in green and Unexpressed” in red. Model specificity (A) was computed as the number of model genes in each category, while model completeness was computed as the ratio of model genes in a category and the total number of genes in that category. Our glioma subtype models showed comparable specificity and completeness compared to the curated and semi-curated models in the brain.


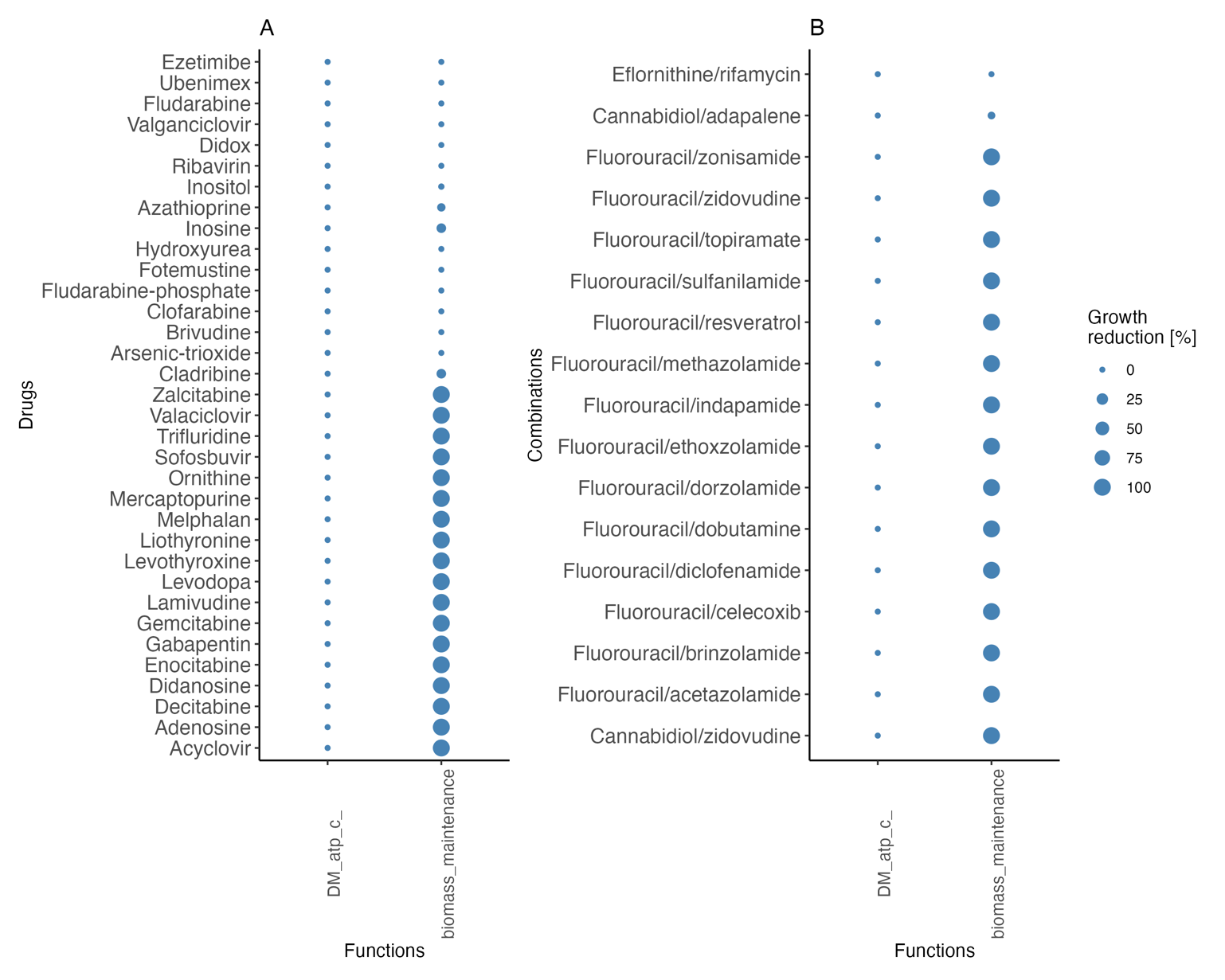


**Figure S12: Half of the single drugs and two combinations show a minimal effect on the healthy biomass maintenance reaction of the control model.**

To determine which of the predicted single drugs and combinations are potentially toxic to the healthy brain, drug deletion was applied to a control brain GEM built from five healthy samples in TCGA-GBM. Growth reduction (1-grRatio) was used to rank the predicted drugs based on potential safety for two reactions: ATP production (DM_atp_c_) and growth (biomass_maintainance). All predicted drugs were predicted to be safe on ATP production. Of the single drugs, half are predicted to be safe for healthy brain growth, including clinically effective drugs: fotemustine and valganciclovir. Meanwhile, the subtype-specific combinations (eflornithine/rifamycin and cannabidiol/adapalene) are the only safe combinations on the control model growth.

**Table S5: Summary of the high- or medium-drug screening databases used to evaluate the predicted drugs.**

See the Resources Table for downloadable sources. PDX: patient-derived xenograft

| **Database** | **Type** | **Tested concentration(s)** | **Number of cell lines/ xenografts** | **Number of tested drugs** | **Number of predicted single drugs** | **Number of predicted combination drugs** | **Reference** |
| --- | --- | --- | --- | --- | --- | --- | --- |
| Stathias et al., 2018 | *In vivo* PDX in mice | 1 μM | 7 (GBM) | 186 | 10 | 3 | ^40^ |
| Bell et al. 2018 Initial screen | *Ex vivo* PDX in mice | 10 μM | 7 (GBM) | 520 | 9 | 2 | ^41^ |
| Bell et al. 2018 follow-up screen | *Ex vivo* PDX in mice | 0.1, 1, 10 µM | 5 (GBM) | 119 | 8 | 2 | ^41^ |
| Primary PRISM | *In vitro* (viability reduction) | 2.5-5 µM | 25 (GBM),10 (AST) | 4517 | 31 | 17 | ^42^ |
| Nam et al. 2021 | *In vitro* (viability reduction) | 10 µM | 2 (GBM) | 975 | 11 | 8 | ^43^ |
| gCSI | *In vitro* (IC_50_) | 0.01-20 µM | 8 (GBM), 5 (AST), 1 (ODG), 1 (non-glioma) | 16 | 1 | 0 | ^44^ |
| GDSC1000 | *In vitro* (IC_50_) | 3.125e-5 to 15.625 μM | 27 (GBM), 9 (AST), 1 (ODG), 4 (non-glioma) | 378 | 1 | 1 | ^45^ |
| GDSC2000 | *In vitro* (IC_50_) | 3.125e-5 to 15.625 μM | 28 (GBM), 9 (AST), 1 (ODG), 4 (non-glioma) | 286 | 1 | 1 | ^45^ |
| Secondary PRISM | *In vitro* (IC_50_) | 6.1e-3 to 10 µM | 21 (GBM), 5 (AST), 2 (non-glioma) | 1414 | 14 | 4 | ^42^ |

gCSI: Genentech Cell Line Screening Initiative

**Table S6: Classification criteria of the predicted single and combination drugs into effective, ineffective, and untested compared to the AntiBCs.**

|  | **Evidence** | **Effective** | **Ineffective** | **Untested** |
| --- | --- | --- | --- | --- |
| **Single drugs** | ***In vitro*** | - Comparable/improved CSF bioavailability/potency to AntiBCs - >50% viability reduction - Induced apoptosis/autophagy | - Induced proliferation - Remaining tested drugs *in vitro* |  |
|  | ***In/ex vivo*** | - >25% growth reduction in GBM xenografts screening databases - Enhanced growth reduction or survival in literature | - Remaining tested drugs *In/ex vivo* |  |
|  | **Clinical trial** | - Phase II, two-arms, with improved OS/PFS as monotherapy /in combination against AntiBCs | - Declared non-effective in single-arm phase II trial - Showed antagonism while combined with AntiBCs in phase II trial | - Remaining drugs, including single-arm phase II trials |
| **Combination drugs** | ***In vitro*** | - In addition to single drugs: - Synergism/additive/ chemosensitizer effect with TMZ | - In addition to single drugs, antagonism effect with TMZ |  |
|  | ***In/ex vivo*** | - In addition to single drugs: - Synergism/additive/ chemosensitizer effect with TMZ - Radio-sensitization | - In addition to single drugs, antagonism effect with TMZ |  |
|  | **Clinical trial** | - Same as single drugs |  |  |


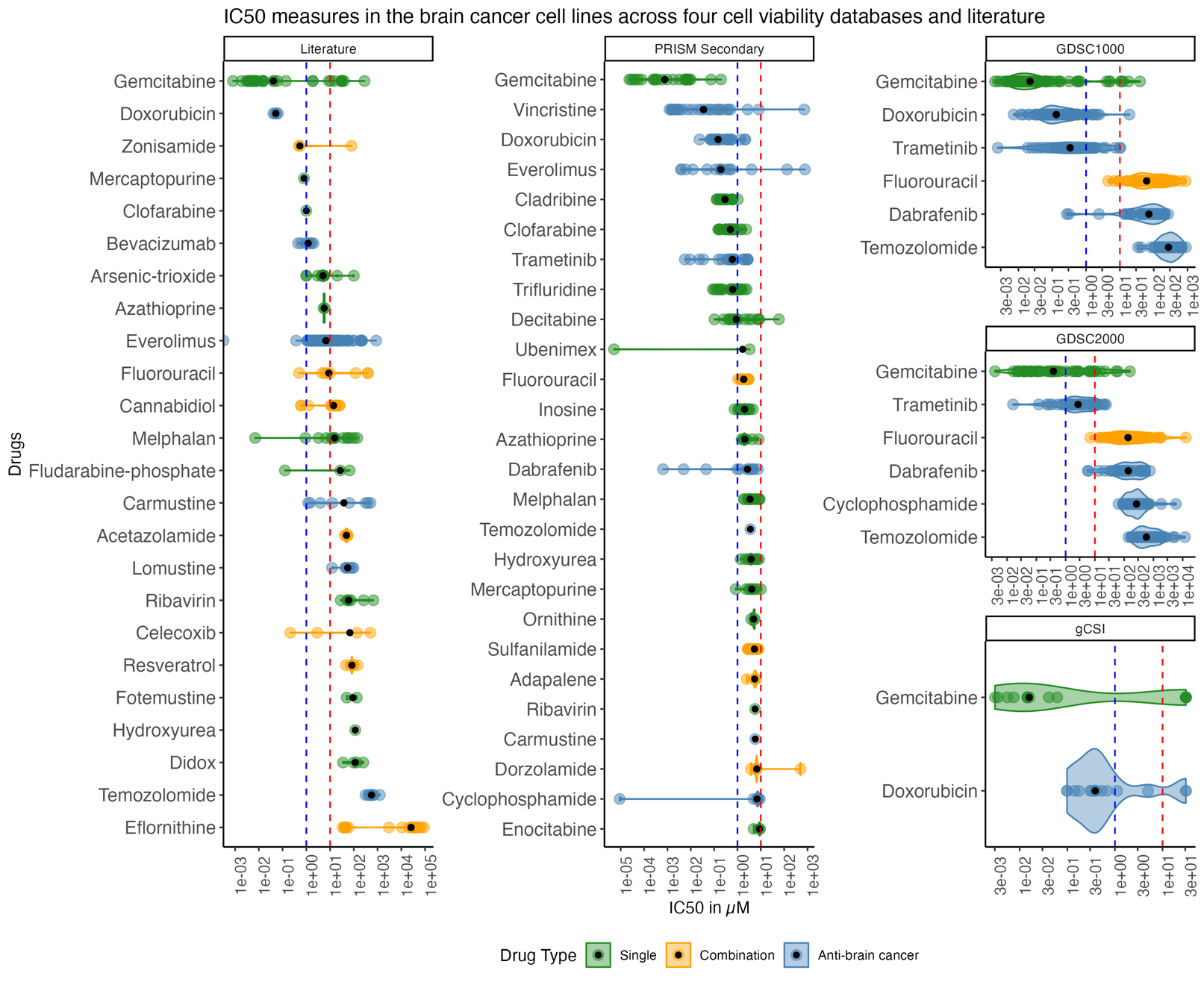


**Figure S13: Gemcitabine showed superior potency in all IC_50_** **databases to approved AntiBCs, while alkylating AntiBCs are commonly used for glioma.**

Drug screening databases with IC_50_ measures and IC_50_ measures collected from literature (**Supplementary File 2, Table S13**) were used to rank predicted single, combinations drugs and AntiBCs using median IC_50_ as a potency measure. Alkylating AntiBCs (temozolomide, lomustine, carmustine, and cyclophosphamide) show the lowest potency, with gemcitabine having the highest potency across the five databases. Other combinations of drugs, such as zonisamide and fluorouracil, showed comparable potency to non-alkylating AntiBCs and higher than alkylating AntiBCs.


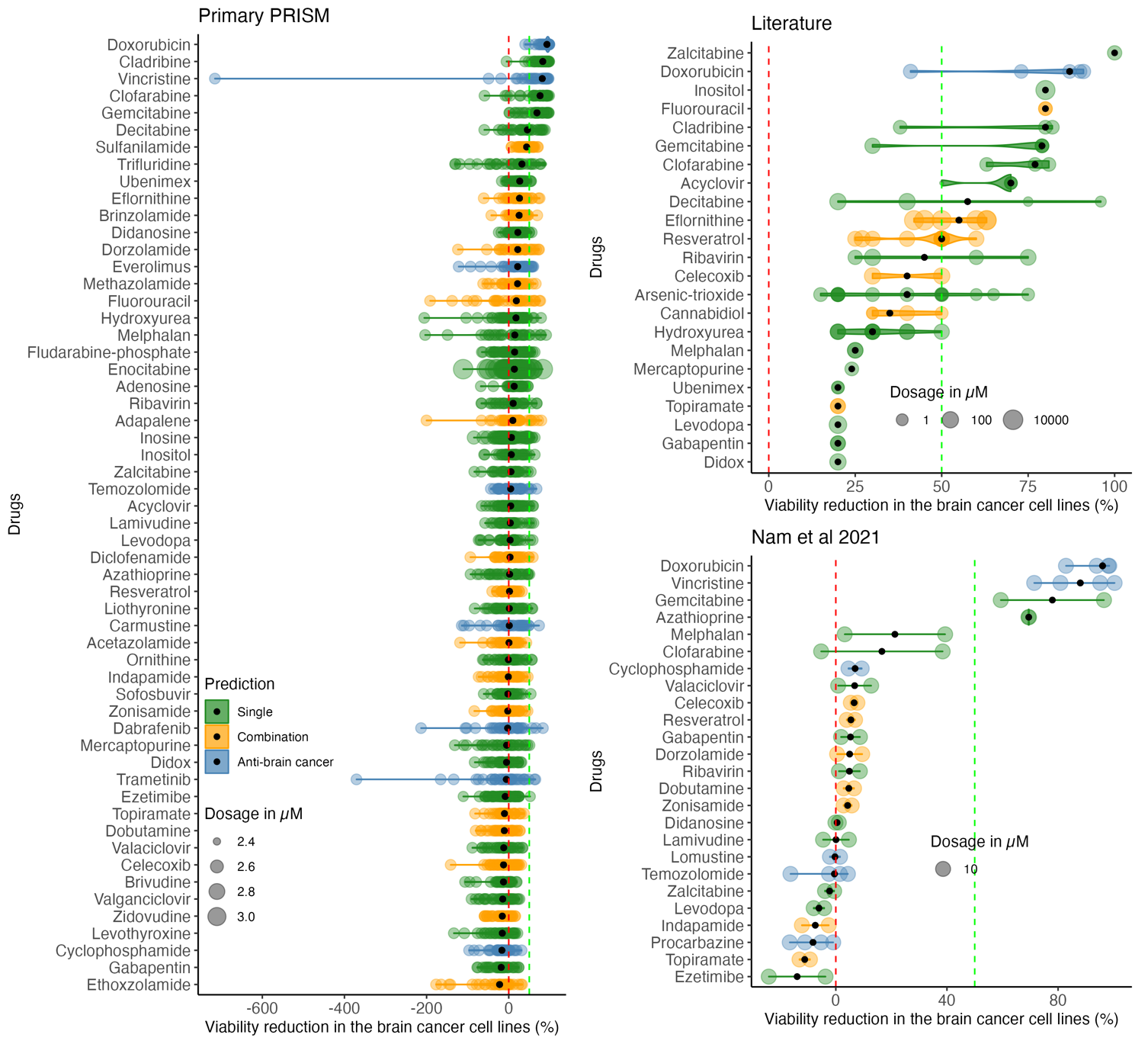


**Figure S14: Doxorubicin, followed by the anti-metabolites, shows the strongest viability reduction in the cell viability databases and literature.**

Besides the IC_50_ data, other drug screening databases included only viability reduction with a small range of tested concentrations. Viability reduction from two databases and literature (**Supplementary File 2, Table S13**) was used to compare the single and combination drugs to AntiBCs using the median viability reduction (black dot). Doxorubicin was the only AntiBCs, followed by primarily anti-metabolites of the single drugs to exceed 50% viability reduction.

**Table S7: Two of three predicted combinations showed synergistic interactions in non-brain cancer cell lines.** Previous combined testing of each drug in a combination is essential to rank the predicted combinations. PubMed and clinicaltrials.gov were searched for any previous testing of the 17 predicted combinations, including non-cancer, in vitro, and clinical trials. None of the combinations were found to be tested clinically in brain cancer. Only three combinations were tested *in vitro* in non-brain cancer with synergistic actions, of which two failed in clinical trials. These tests were used to rank the combinations (**Supplementary File 2, Table S10**).

| **Predicted combination** | **Clinical /Preclinical** | **Cancer type** | **The rationale for testing this combination** | **Result** | **Side effect (if exists)** | **Reference** |
| --- | --- | --- | --- | --- | --- | --- |
| Fluorouracil/zidovudine | Preclinical | Colon cancer (HT-29) |  | Synergistic effect |  | ^46^ |
| Fluorouracil/zidovudine | Clinical (Phase I, n=18) | Various tumors | Preclinical synergistic cytotoxic effect | Terminated, as the plasma concentration of the anti-cancer activity of zidovudine is above the maximal dose |  | ^47^ |
| Fluorouracil/celecoxib | Preclinical | Gastric carcinoma cell line | Fluorouracil resistance in gastric cancer is correlated with COX-2 expression | Synergistic effect | Celecoxib did not have additional toxicity to fluorouracil | ^48^ |
| Fluorouracil/celecoxib | Clinical (Phase III, n= 2526) | Colon Cancer (Stage III) | Inhibiting COX2 might enhance OS | There is no notable change in OS compared to fluorouracil alone | Tolerated; a slight increase in hypertension | ^49^ |
| Fluorouracil/resveratrol | Preclinical | Colon cancer (HCT116, DLD1) |  | Resveratrol increased the anti-telomeric and pro-apoptotic actions of fluorouracil |  | ^50^ |
| Fluorouracil/resveratrol | Preclinical | Normal human keratinocytes (HaCaT) | Resveratrol may avoid fluorouracil-induced side effects | Resveratrol reduced fluorouracil-induced ROS production |  | ^51^ |

**Resources table**

| **Resources** | **Version** | **Link** |
| --- | --- | --- |
| **RNA-Seq of TCGA-GBM and TCGA-LGG** |  | https://www.ncbi.nlm.nih.gov/geo/query/acc.cgi?acc=GSE62944 |
| **List of common essential genes** | DepMap 22Q1 | https://ndownloader.figshare.com/files/34008470 |
| **Stathias et al 2018 GBM PDX data** |  | https://data.mendeley.com/datasets/yz8m28gj6r/1 |
| **Bell et al. 2018, initial screen on GBM PDXs** |  | <https://doi.org/10.1158/1541-7786.MCR-17-0397>, Supplementary Table 1 |
| **Bell et al. 2018, follow-up screen on GBM PDXs** |  | <https://doi.org/10.1158/1541-7786.MCR-17-0397>, Supplementary Table 2 |
| **Primary and secondary PRISM screen** | PRISM Repurposing 19Q4 | https://depmap.org/portal/download/all/ |

**References:**

1. Louis DN, Perry A, Wesseling P, et al. The 2021 WHO classification of tumors of the central nervous system: A summary. *Neuro Oncol*. 2021;23(8):1231-1251. doi:10.1093/NEUONC/NOAB106

2. Weinstein JN, Collisson EA, Mills GB, et al. The cancer genome atlas pan-cancer analysis project. *Nat Genet*. 2013;45(10). doi:10.1038/ng.2764

3. Colaprico A, Silva TC, Olsen C, et al. TCGAbiolinks: an R/Bioconductor package for integrative analysis of TCGA data. *Nucleic Acids Res*. 2016;44(8):e71. doi:10.1093/NAR/GKV1507

4. Thiele I, Swainston N, Fleming RMT, et al. A community-driven global reconstruction of human metabolism. *Nat Biotechnol*. 2013;31(5):419-425. doi:10.1038/nbt.2488

5. Brunk E, Sahoo S, Zielinski DC, et al. Recon3D enables a three-dimensional view of gene variation in human metabolism. *Nat Biotechnol*. 2018;36(3):272-281. doi:10.1038/nbt.4072

6. Robinson JL, Kocabaş P, Wang H, et al. An atlas of human metabolism. *Sci Signal*. 2020;13(624). doi:10.1126/scisignal.aaz1482

7. Rahman M, Jackson LK, Johnson WE, Li DY, Bild AH, Piccolo SR. Alternative preprocessing of RNA-Sequencing data in The Cancer Genome Atlas leads to improved analysis results. *Bioinformatics*. 2015;31(22):3666-3672. doi:10.1093/bioinformatics/btv377

8. Ceccarelli M, Barthel FP, Malta TM, et al. Molecular Profiling Reveals Biologically Discrete Subsets and Pathways of Progression in Diffuse Glioma. *Cell*. 2016;164(3):550-563. doi:10.1016/J.CELL.2015.12.028

9. Mounir M, Lucchetta M, Silva TC, et al. New functionalities in the TCGAbiolinks package for the study and integration of cancer data from GDC and GTEX. *PLoS Comput Biol*. 2019;15(3). doi:10.1371/journal.pcbi.1006701

10. Wishart DS, Feunang YD, Marcu A, et al. HMDB 4.0: the human metabolome database for 2018. *Nucleic Acids Res*. 2018;46(Database issue):D608-D617. doi:10.1093/nar/gkx1089

11. Thiele I, Sahoo S, Heinken A, et al. Personalized whole‐body models integrate metabolism, physiology, and the gut microbiome. *Mol Syst Biol*. 2020;16(5):e8982. doi:10.15252/msb.20198982

12. Pacheco MP, Bintener T, Ternes D, et al. Identifying and targeting cancer-specific metabolism with network-based drug target prediction. *EBioMedicine*. 2019;43:98-106. doi:10.1016/J.EBIOM.2019.04.046

13. Pacini C, Dempster JM, Boyle I, et al. Integrated cross-study datasets of genetic dependencies in cancer. *Nat Commun*. 2021;12(1). doi:10.1038/S41467-021-21898-7

14. Meng F, Xi Y, Huang J, Ayers PW. A curated diverse molecular database of blood-brain barrier permeability with chemical descriptors. *Sci Data*. 2021;8(1). doi:10.1038/s41597-021-01069-5

15. Siramshetty VB, Grishagin I, Nguyen DT, et al. NCATS Inxight Drugs: A comprehensive and curated portal for translational research. *Nucleic Acids Res*. 2022;50(D1). doi:10.1093/nar/gkab918

16. Jacobs AH, Thomas A, Kracht LW, et al. 18F-Fluoro-l-Thymidine and 11C-Methylmethionine as Markers of Increased Transport and Proliferation in Brain Tumors. *Journal of Nuclear Medicine*. 2005;46(12).

17. Nikaki A, Angelidis G, Efthimiadou R, et al. 18F-fluorothymidine PET imaging in gliomas: an update. *Ann Nucl Med*. 2017;31(7):495. doi:10.1007/S12149-017-1183-2

18. Chiu M, Taurino G, Bianchi MG, et al. Oligodendroglioma Cells Lack Glutamine Synthetase and Are Auxotrophic for Glutamine, but Do not Depend on Glutamine Anaplerosis for Growth. *International Journal of Molecular Sciences 2018, Vol 19, Page 1099*. 2018;19(4):1099. doi:10.3390/IJMS19041099

19. Mörén L, Tommy Bergenheim A, Ghasimi S, Brännström T, Johansson M, Antti H. Metabolomic Screening of Tumor Tissue and Serum in Glioma Patients Reveals Diagnostic and Prognostic Information. *Metabolites 2015, Vol 5, Pages 502-520*. 2015;5(3):502-520. doi:10.3390/METABO5030502

20. Ganji SK, An Z, Tiwari V, et al. In Vivo Detection of 2-Hydroxyglutarate in Brain Tumors by Optimized PRESS at 7T. *Magn Reson Med*. 2017;77(3):936. doi:10.1002/MRM.26190

21. Pitt D, Nagelmeier IE, Wilson HC, Raine CS. Glutamate uptake by oligodendrocytes: Implications for excitotoxicity in multiple sclerosis. *Neurology*. 2003;61(8):1113-1120. doi:10.1212/01.WNL.0000090564.88719.37

22. Chaumeil MM, Radoul M, Najac C, et al. Hyperpolarized 13C MR imaging detects no lactate production in mutant IDH1 gliomas: Implications for diagnosis and response monitoring. *Neuroimage Clin*. 2016;12. doi:10.1016/j.nicl.2016.06.018

23. Jovanović M, Dragoj M, Zhukovsky D, et al. Novel TrxR1 Inhibitors Show Potential for Glioma Treatment by Suppressing the Invasion and Sensitizing Glioma Cells to Chemotherapy. *Front Mol Biosci*. 2020;7:281. doi:10.3389/FMOLB.2020.586146/BIBTEX

24. Pires V, Bramatti I, Aschner M, Branco V, Carvalho C. Thioredoxin Reductase Inhibitors as Potential Antitumors: Mercury Compounds Efficacy in Glioma Cells. *Front Mol Biosci*. 2022;9. doi:10.3389/FMOLB.2022.889971

25. Zhang Y, Chen F, Tai G, et al. TIGAR knockdown radiosensitizes TrxR1-overexpressing glioma in vitro and in vivo via inhibiting Trx1 nuclear transport. *Scientific Reports 2017 7:1*. 2017;7(1):1-13. doi:10.1038/srep42928

26. Haapasalo H, Kyläniemi M, Paunu N, Kinnula VL, Soini Y. Expression of Antioxidant Enzymes in Astrocytic Brain Tumors. *Brain Pathology*. 2003;13(2):155-164. doi:10.1111/J.1750-3639.2003.TB00015.X

27. Hannu H, Helena B, Niina P, et al. Antioxidant enzymes in oligodendroglial brain tumors: Association with proliferation, apoptotic activity and survival. *J Neurooncol*. 2006;77(2):131-140. doi:10.1007/S11060-005-9030-Z/METRICS

28. Sun P, Xia S, Lal B, et al. Lipid metabolism enzyme ACSVL3 supports glioblastoma stem cell maintenance and tumorigenicity. *BMC Cancer*. 2014;14(1). doi:10.1186/1471-2407-14-401

29. Teng J, Hejazi S, Hiddingh L, et al. Recycling drug screen repurposes hydroxyurea as a sensitizer of glioblastomas to temozolomide targeting de novo DNA synthesis, irrespective of molecular subtype. *Neuro Oncol*. 2018;20(5):642-654. doi:10.1093/NEUONC/NOX198

30. Ariey-Bonnet J, Berges R, Montero MP, et al. Combination drug screen targeting glioblastoma core vulnerabilities reveals pharmacological synergisms. *EBioMedicine*. 2023;95. doi:10.1016/j.ebiom.2023.104752

31. Bernhart E, Damm S, Wintersperger A, et al. Interference with distinct steps of sphingolipid synthesis and signaling attenuates proliferation of U87MG glioma cells. *Biochem Pharmacol*. 2015;96(2):119-130. doi:10.1016/J.BCP.2015.05.007

32. Chen A, Zhao W, Li X, et al. Comprehensive Oncogenic Features of Coronavirus Receptors in Glioblastoma Multiforme. *Front Immunol*. 2022;13. doi:10.3389/fimmu.2022.840785

33. Pavlyk I, Rzhepetskyy Y, Jagielski AK, et al. Arginine deprivation affects glioblastoma cell adhesion, invasiveness and actin cytoskeleton organization by impairment of β-actin arginylation. *Amino Acids*. 2015;47(1). doi:10.1007/s00726-014-1857-1

34. Sun Y, Wang C, Wang L, Dai Z, Yang K. Arsenic trioxide induces apoptosis and the formation of reactive oxygen species in rat glioma cells. *Cell Mol Biol Lett*. 2018;23(1):1-10. doi:10.1186/S11658-018-0074-4/FIGURES/5

35. Said HM, Hagemann C, Carta F, et al. Hypoxia induced CA9 inhibitory targeting by two different sulfonamide derivatives including Acetazolamide in human Glioblastoma. *Bioorg Med Chem*. 2013;21(13):3949-3957. doi:10.1016/J.BMC.2013.03.068

36. Massi P, Vaccani A, Bianchessi S, Costa B, Macchi P, Parolaro D. The non-psychoactive cannabidiol triggers caspase activation and oxidative stress in human glioma cells. *Cellular and Molecular Life Sciences*. 2006;63(17). doi:10.1007/s00018-006-6156-x

37. Khan A, Gamble LD, Upton DH, et al. Dual targeting of polyamine synthesis and uptake in diffuse intrinsic pontine gliomas. *Nat Commun*. 2021;12(1). doi:10.1038/s41467-021-20896-z

38. Kishk A, Pacheco MP, Heurtaux T, et al. Review of Current Human Genome-Scale Metabolic Models for Brain Cancer and Neurodegenerative Diseases. *Cells*. 2022;11(16). doi:10.3390/CELLS11162486/S1

39. Sjöstedt E, Zhong W, Fagerberg L, et al. An atlas of the protein-coding genes in the human, pig, and mouse brain. *Science (1979)*. 2020;367(6482). doi:10.1126/science.aay4106

40. Stathias V, Jermakowicz AM, Maloof ME, et al. Drug and disease signature integration identifies synergistic combinations in glioblastoma. *Nature Communications 2018 9:1*. 2018;9(1):1-13. doi:10.1038/s41467-018-07659-z

41. Bell JB, Eckerdt F, Dhruv HD, et al. Differential response of glioma stem cells to arsenic trioxide therapy is regulated by MNK1 and mRNA translation. *Molecular Cancer Research*. 2018;16(1). doi:10.1158/1541-7786.MCR-17-0397

42. Corsello SM, Nagari RT, Spangler RD, et al. Discovering the anti-cancer potential of non-oncology drugs by systematic viability profiling. *Nat Cancer*. 2020;1(2):235-248. doi:10.1038/s43018-019-0018-6

43. Nam HJ, Kim YE, Moon BS, et al. Azathioprine antagonizes aberrantly elevated lipid metabolism and induces apoptosis in glioblastoma. *iScience*. 2021;24(3). doi:10.1016/j.isci.2021.102238

44. Klijn C, Durinck S, Stawiski EW, et al. A comprehensive transcriptional portrait of human cancer cell lines. *Nat Biotechnol*. 2015;33(3). doi:10.1038/nbt.3080

45. Yang W, Soares J, Greninger P, et al. Genomics of Drug Sensitivity in Cancer (GDSC): a resource for therapeutic biomarker discovery in cancer cells. *Nucleic Acids Res*. 2013;41(D1):D955—-D961. doi:10.1093/nar/gks1111

46. Weber G, Ichikawa S, Nagai M, Natsumeda Y. Azidothymidine inhibition of thymidine kinase and synergistic cytotoxicity with methotrexate and 5-fluorouracil in rat hepatoma and human colon cancer cells. *Cancer Commun*. 1990;2(4). doi:10.3727/095535490820874498

47. Beitz JG, Damowski JW, Cummings FJ, et al. Phase I trial of high-dose infused zidovudine combined with leucovorin plus fluorouracil. *Cancer Invest*. 1995;13(5). doi:10.3109/07357909509024908

48. Choi SM, Cho YS, Park G, Lee SK, Chun KS. Celecoxib induces apoptosis through Akt inhibition in 5-fluorouracil-resistant gastric cancer cells. *Toxicol Res*. 2021;37(1). doi:10.1007/s43188-020-00044-3

49. Meyerhardt JA, Shi Q, Fuchs CS, et al. Effect of Celecoxib vs Placebo Added to Standard Adjuvant Therapy on Disease-Free Survival among Patients with Stage III Colon Cancer: The CALGB/SWOG 80702 (Alliance) Randomized Clinical Trial. *JAMA - Journal of the American Medical Association*. 2021;325(13). doi:10.1001/jama.2021.2454

50. Chung SS, Dutta P, Austin D, Wang P, Awad A, Vadgama J V. Combination of resveratrol and 5-flurouracil enhanced antitelomerase activity and apoptosis by inhibiting STAT3 and Akt signaling pathways in human colorectal cancer cells. *Oncotarget*. 2018;9(68). doi:10.18632/oncotarget.25993

51. Chen S, Tamaki N, Kudo Y, et al. Protective effects of resveratrol against 5-fluorouracil-induced oxidative stress and inflammatory responses in human keratinocytes. *J Clin Biochem Nutr*. 2021;69(3). doi:10.3164/jcbn.21-23
